# Sequential filtering for clinically relevant variants as a method for clinical interpretation of whole exome sequencing findings in glioma

**DOI:** 10.1186/s12920-021-00904-3

**Published:** 2021-02-23

**Authors:** Ege Ülgen, Özge Can, Kaya Bilguvar, Cemaliye Akyerli Boylu, Şirin Kılıçturgay Yüksel, Ayça Erşen Danyeli, O. Uğur Sezerman, M. Cengiz Yakıcıer, M. Necmettin Pamir, Koray Özduman

**Affiliations:** 1 Department of Biostatistics and Medical Informatics, School of Medicine, Acibadem Mehmet Ali Aydinlar University, Istanbul, Turkey; 2 Department of Medical Engineering, Faculty of Engineering, Acibadem Mehmet Ali Aydinlar University, Istanbul, Turkey; 3 Department of Genetics, School of Medicine, Yale University, New Haven, CT USA; 4 Yale Center for Genome Analysis, West Haven, CT USA; 5 Department of Medical Biology, School of Medicine, Acibadem Mehmet Ali Aydinlar University, Istanbul, Turkey; 6 Department of Pathology, School of Medicine, Acibadem Mehmet Ali Aydinlar University, Istanbul, Turkey; 7 Department of Molecular Biology, School of Arts and Sciences, Acibadem Mehmet Ali Aydinlar University Istanbul, Istanbul, Turkey; 8 Department of Neurosurgery, School of Medicine, Acibadem Mehmet Ali Aydinlar University, Altunizade Mahallesi, Yurtcan Sok. No:1, Üsküdar, Istanbul, 34662 Turkey

**Keywords:** Whole exome sequencing, NGS, Glioma, Brain tumor, Clinical analysis

## Abstract

**Background:**

In the clinical setting, workflows for analyzing individual genomics data should be both comprehensive and convenient for clinical interpretation. In an effort for comprehensiveness and practicality, we attempted to create a clinical individual whole exome sequencing (WES) analysis workflow, allowing identification of genomic alterations and presentation of neurooncologically-relevant findings.

**Methods:**

The analysis workflow detects germline and somatic variants and presents: (1) germline variants, (2) somatic short variants, (3) tumor mutational burden (TMB), (4) microsatellite instability (MSI), (5) somatic copy number alterations (SCNA), (6) SCNA burden, (7) loss of heterozygosity, (8) genes with double-hit, (9) mutational signatures, and (10) pathway enrichment analyses. Using the workflow, 58 WES analyses from matched blood and tumor samples of 52 patients were analyzed: 47 primary and 11 recurrent diffuse gliomas.

**Results:**

The median mean read depths were 199.88 for tumor and 110.955 for normal samples. For germline variants, a median of 22 (14–33) variants per patient was reported. There was a median of 6 (0–590) reported somatic short variants per tumor. A median of 19 (0–94) broad SCNAs and a median of 6 (0–12) gene-level SCNAs were reported per tumor. The gene with the most frequent somatic short variants was *TP53* (41.38%). The most frequent chromosome-/arm-level SCNA events were chr7 amplification, chr22q loss, and chr10 loss. TMB in primary gliomas were significantly lower than in recurrent tumors (*p* = 0.002). MSI incidence was low (6.9%).

**Conclusions:**

We demonstrate that WES can be practically and efficiently utilized for clinical analysis of individual brain tumors. The results display that NOTATES produces clinically relevant results in a concise but exhaustive manner.

## Background

Next-generation sequencing (NGS) has proven remarkably beneficial in not only understanding cancer biology but also guiding cancer care [1–3]. Various NGS methods are routinely used in cancer care [4, 5]. Targeted sequencing panels, whole-exome sequencing (WES), and whole-genome sequencing (WGS) are the most commonly utilized methods, each with its advantages and limitations [6–8]. Targeted sequencing panels are tailored to investigate curated cancer-related information, provide excellent depth, and are suited for working with formalin-fixed paraffin-embedded (FFPE) samples [9, 10]. In contrast, WES/WGS provides more comprehensive genomics data suited for both screening previously investigated/reported variants and exploring novel relevant variants. More comprehensive genomics data also provide additional information such as direct measurement of the mutational burden [11, 12] and exploration of signatures of mutational processes [13, 14]. Brain tumors have complex genetic landscapes [15–17]. Therefore, it is beneficial to gather the most comprehensive genomics information for each neurooncology patient. We hence advocate utilizing WES for neurooncological genomics analyses as it gathers comprehensive information with a lower cost than WGS and is technically less challenging to analyze and interpret.

The bioinformatics workflows for variant calling are well established but the clinical interpretation of the identified variants constitutes a bottleneck in the analysis [18]. In the clinical setting, the analysis workflow should produce results that are both exhaustive and suitable for clinical interpretation. Intending to be simultaneously comprehensive and practical, we created a clinical WES workflow tailored for neurooncology. This approach sequentially filters and presents layers of findings relevant to neurooncology (the layers being alterations that are detected in curated collections of clinically-relevant genes). This sequential filtering approach prioritizes highly relevant findings while still reporting less relevant but possibly important findings. This article presents our approach and provides results of the analysis of our findings on a sizable glioma cohort, demonstrating that our approach yields clinically relevant results.

## Methods

### Reads-to-variants workflow

**Fig. 1:**
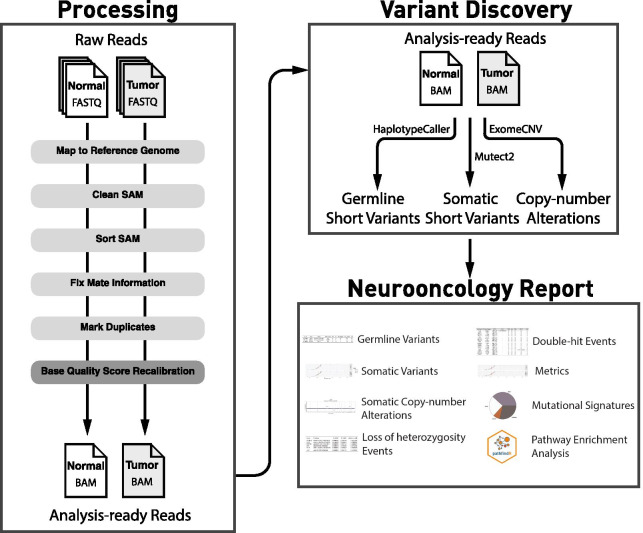
Outline of the reads-to-personalized-report workflow

The overview of the complete workflow is presented in Fig. 1. The reads-to-variants pipeline is presented below.

For quality control, FASTQC (v0.11.9) [19] is used. For tumor and normal samples, the reads are mapped to the reference (hg38) using bwa (version 0.7.17-r1188) [20] and pre-processed, including cleaning the SAM file, sorting SAM by coordinate and converting to BAM, fixing mate information, and marking PCR duplicates (all via Picard version 2.23.8) [21]. For samples that were sequenced in multiple lanes, data for all lanes are combined at this step. Finally, base quality score recalibration (GATK [22] v4.1.9.0) is performed. For quality control, GATK3–DepthOfCoverage (version 3.8-1-0-gf15c1c3ef) and Picard-CollectAlignmentSummaryMetrics are used.

For detecting germline variants (single nucleotide variants (SNVs) and short insertion/deletions (indels)), GATK–HaplotypeCaller is used. For detecting somatic SNV/indels, GATK–MuTect2 is used. Both germline and somatic SNV/indels are annotated using GATK–Funcotator. For detecting somatic copy number alterations (SCNAs), ExomeCNV is used [23]. Annotations of gene-level SCNAs and cytoband annotations are performed via an in-house script.

### Personalized neurooncology report workflow

To produce comprehensive reports of WES results, we developed the reporting workflow NOTATES. NOTATES uses curated datasets of glioma- and cancer-related variants and genes to sequentially report clinically relevant findings.

After a summary of somatic WES findings, the report contains the following sections:

1. Quality Metrics
  - Summary Table of Quality Metrics
  - Tumor Purity
2. Germline Alterations
  - ACMG Incidental Findings
  - Variations in Cancer Gene Census Genes
  - Variations in Cancer Predisposition Genes
  - Variations in DNA Damage Repair Genes
  - Common Variants
3. Somatic Single Nucleotide Variations (SNVs) and Small Insertion/Deletions (Indels)
  - Tumor Mutational Burden (TMB)
  - Microsatellite Instability Status (MSI)
  - Variants in Established Glioma Genes
  - Hotspot Variants in Cancer Gene Census Genes
  - Other Variants in Cancer Gene Census Genes
  - Other Possibly Important Somatic SNV/indels
    i. Variants in DNA Damage Repair Genes
    ii. Variants in Important KEGG Pathway Genes
4. Somatic Copy Number Alterations (SCNAs)
  - SCNA Burden
  - Established SCNAs in Glioma
  - SCNAs in Cancer Gene Census Genes
  - Broad SCNAs
  - Plots of SCNA Segments by Chromosome
5. Loss of Heterozygosity (LOH) Events
  - LOH Overview
  - LOH + Somatic SNV/Indel
  - LOH Events in Cancer Gene Census Genes
6. Genes with Double Hit
7. Tumor Heterogeneity Analysis
8. Mutational Signatures
9. pathfindR—KEGG Pathway Enrichment Analysis

The contents of these sections are detailed in the Results section. NOTATES was written in R [24] and R Markdown.

### Analyses and patients

Using NOTATES v1.5, 58 WES analyses from matched blood and tumor samples of 52 patients were analyzed: 47 primary and 11 recurrent diffuse gliomas. Overall, 47 grade IV (81.03%), 7 grade III (12.07%), and 4 grade II tumors (6.9%) were analyzed. Clinical details for all patients and analyses are presented in Additional file 2: Table S1. For each tumor specimen submitted for WES, sections were reviewed by a neuro-pathologist to confirm the diagnosis of diffuse glioma and specifically excise a region within the tumor sample containing only tumor tissue. DNA was extracted using the DNeasy Blood & Tissue Kit (QIAGEN).

All analyses of NOTATES results presented here were performed using R. Selected results were compared with results from the TCGA pan-glioma cohort [15].

### Software availability

The reads-to-variants and reporting workflow NOTATES is available for non-commercial purposes on GitHub: https://github.com/egeulgen/NOTATES.

## Results

### Analysis and reporting of exomes

#### Sequencing quality metrics

The median mean read depths were 199.88 for tumor and 110.955 for normal samples. The median percentages of reads with at least 25X coverage were 99.3% and 98.55% for tumor and normal samples, respectively. Detailed quality metrics are presented in Additional file 2: Table S2.

#### Germline variants

**Fig. 2:**
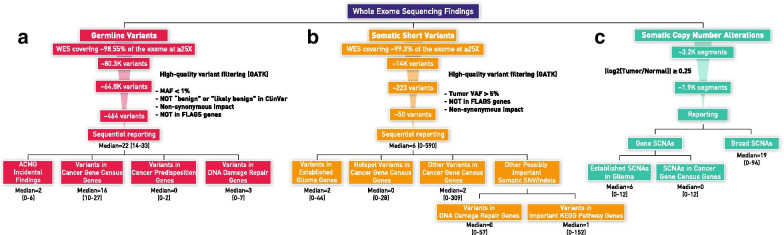
Variant analysis and reporting workflow for **a** germline variants, **b** somatic short variants, and **c** somatic copy number alterations

Raw germline variants (median = 80,328, range = 72,008–120,635 per patient) are initially filtered according to GATK's best practices [22] for eliminating technical artifacts to yield a median of 64,815 (range = 58,528–87,619) variants per patient (Fig. 2a). For reporting, we only include variants that:

- have MAF < 1%
- are not reported as “benign” or “likely benign” in ClinVar [25]
- have non-synonymous impact
- are not in FLAGS [26] genes.

This filtering results in a median of 464 (range = 400–536) variants per patient. A median of 22 (range = 14–33) variants per patient is in the reported categories: A median of 2 (range = 0–6) in “ACMG Incidental Findings”, 16 (range = 10–27) “Variants in Cancer Gene Census Genes”, 0 (range = 0–2) in “Variants in Cancer Predisposition Genes” and 3 (range = 0–7) in “Variants in DNA Damage Repair Genes”.

Considerable percentages of combined reported variants (in all patients) per each category did not have a record in ClinVar (“not reported”) and for variants with a ClinVar record. The most frequent clinical significances were “Drug response” for “ACMG Incidental Findings” (37.3%), “Conflicting” for “Variants in Cancer Gene Census Genes” (5.17%), and “VUS” for “Variants in Cancer Predisposition Genes” (16.67%) and “Variants in DNA Damage Repair Genes” (3.82%) (Additional file 2: Fig. S1). Very small fractions of reported variants per each category were reported as “Pathogenic” or “Likely Pathogenic”: 2.38% for “ACMG Incidental Findings”, 0.48% for “Variants in Cancer Gene Census Genes”, 4.17% for “Variants in Cancer Predisposition Genes” and 1.91% for “Variants in DNA Damage Repair Genes” (Additional file 2: Fig. S1).

#### Somatic short variants

To filter out sequencing artifacts, raw somatic short variants (median = 14,000, range = 4068–55,533 per analysis) are similarly filtered following the GATK best practices recommendations to result in a median of 223 (range = 57–22,271) variants per analysis (Fig. 2b). For reporting, we further filter these “called” variants and only include variants that:

- have tumor Variant Allele Frequency (VAF) > 5%
- have non-synonymous impact
- are not in FLAGS genes.

This filtering results in a median of 49.5 (range = 2–5646) variants per analysis. A median of 6 (range = 0–590) variants is in the reported categories: A median of 2 (range = 0–44) in “Variants in Established Glioma Genes”, 0 (range = 0–28) in “Hotspot Variants in Cancer Gene Census Genes”, 2 (0–309) in “Other Variants in Cancer Gene Census Genes”, 0 (range = 0–57) in “Variants in DNA Damage Repair Genes” and 1 (range = 0–152) in “Variants in Important KEGG Pathway Genes”.

**Fig. 3:**
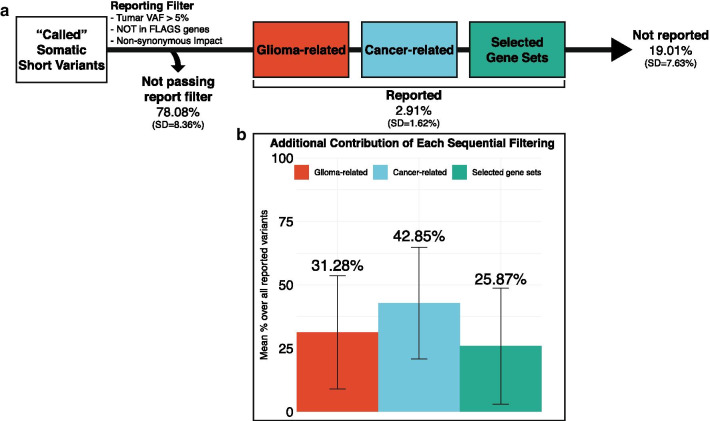
Sequential reporting of called somatic short variants. **a** Flow diagram displaying the sequential reporting process of “called” (technical artifacts excluded) somatic short variants. Mean (standard deviation) percentages over all “called” somatic short variants are indicated. **b** Bar plot displaying mean percentage of somatic short variants reported in each category over all reported variants. Error bars indicate standard deviation

Figure 3 presents the reasoning behind the sequential filtering of somatic short variants. “Called” (sequencing artifacts excluded) somatic short variants are initially filtered according to the above-mentioned criteria, excluding an average of 78.08% (SD = 8.36%) of “called” variants (Fig. 3a). An average of 2.91% (SD = 1.62%) of “called” variants were reported sequentially in the (1) “Glioma-related” subsection (“Variants in Established Glioma Genes”), (2) “Cancer-related” subsections (“Hotspot Variants in Cancer Gene Census Genes” and “Other Variants in Cancer Gene Census Gene”) and (3) “Selected Gene Sets” subsections (“Variants in DNA Damage Repair Genes” and “Variants in Important KEGG Pathway Genes”). On average, 19.01% (SD = 7.63%) did pass the reporting filter but were not reported. By sequential filtering, a variant reported in a category is not reported in the following ones. A mean percentage of 31.28% (SD = 22.37%) of all reported short somatic variants were in the “Glioma-related” subsection, 42.85% (SD = 21.99%) were in the “Cancer-related” subsections and 25.87% (SD = 22.92%) were in “Selected Gene Sets” subsections (Fig. 3b).

#### Somatic copy number alterations

ExomeCNV analysis yields a median of 3222 (range = 112–42,370) segments per analysis (Fig. 2C). For high confidence, only SCNAs with a |log_2_(Tumor/Normal) ratio|≥ 0.25 are reported (median = 1964, range = 66–26,636 segments per analysis). For gene SCNA events, under “Established SCNAs in Glioma”, a median of 6 (range = 0–12) SCNA events per analysis are reported, and a median of 0 (range = 0–12) SCNA events per analysis are reported under “SCNAs in Cancer Gene Census Genes”. Under “Broad SCNAs” a median of 19 (range = 0–94) cytoband-level SCNA events per analysis are reported. Chromosomal-arm-level SCNA events in each tumor are presented in Additional file 2: Fig. S2.

#### Tumor mutational burden and microsatellite instability

The TMB values of all tumors are presented in Additional file 2: Fig. S3A. TMB in primary gliomas (median = 3.2/Mb) were significantly lower than the TMB in recurrent cases (median = 5.8/Mb. Wilcoxon, *p* = 0.002). The TMB values in different molecular subsets (devised based on WES findings) were also significantly different (Kruskal–Wallis, p = 0.0072. Additional file 2: Fig. S3B).

The TMB distribution of this glioma cohort was comparable to (i.e., not significantly different than) the TMB distributions of the TCGA–Glioblastoma multiforme (GBM) and TCGA-Low-grade Glioma (LGG) cohorts (t-test p = 0.7 and p = 0.37 for GBM and LGG, respectively. Additional file 2: Fig. S3C).

There were 4 cases (6.9%) that were predicted to have microsatellite instability and none of the cases were predicted to have *POLE* deficiency.

### The most frequently reported alterations

#### Germline variants

**Fig. 4:**
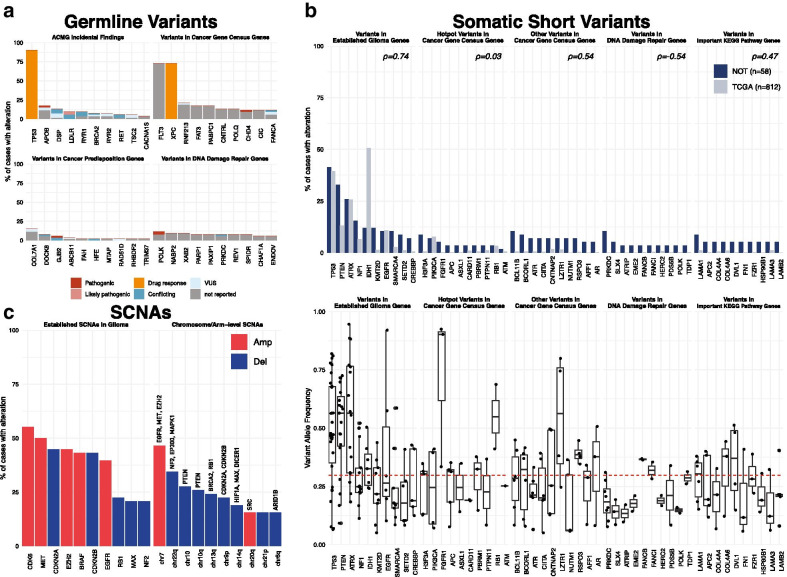
Top 10 genes reported in each subsection. **a** Bar plot displaying the percentages of patients with germline SNV/indel for the top 10 genes in each germline subsection, colored by clinical significance in ClinVar. For patients with multiple germline variants in a given gene, the most severe clinical (as ranked in the legend) significance was used. **b** Bar plot displaying the percentages of analyses with somatic SNV/indel for the top 10 genes in each somatic short variant subsection in the NOT (dark blue) and the TCGA (light blue) cohorts (top) and boxplots displaying the distributions of variant allele frequencies for the same top 10 genes (bottom), red dashed line indicates the median VAF value of all reported variants. **c** Bar plot displaying the percentages of analyses with SCNA events in the top 10 genes in “Established SCNAs in Glioma” (left), and top 10 chromosome-/arm-level SCNAs (right), colored by SCNA type (amplification – “Amp” or deletion – “Del”). Selected consensus tumor suppressor genes/oncogenes located within corresponding genomic regions are indicated

The top 10 genes that harbored a reported germline variant in each subsection are presented in Fig. 4a. A large proportion variants reported in the germline variants section had no clinical significance annotation in ClinVar.

The gene with the most frequent reported germline variants under “ACMG Incidental Findings” was *TP53* with a single variant rs1042522 (“Drug response” clinical significance for antineoplastic agents response in ClinVar, 92.31% of patients). Under “Variants in Cancer Gene Census Genes”, the genes with the most frequent reported variants were *FLT3* with the variant rs1933437 (clinical significance not reported in ClinVar, 73.08%) and *XPC* with the variants with the variant rs2228001 (“Drug response” clinical significance for cisplatin response—Toxicity/ADR in ClinVar, 73.08%). The gene with most frequent reported germline variants under “Variants in Cancer Predisposition Genes” was *COL7A1* with 7 different variants: g.chr3:48569407G>C (not reported, 1.92%), g.chr3:48591687G>T (not reported, 1.92%), rs200868430 (not reported, 1.92%), rs141787797 (not reported, 1.92%), rs116005007 (VUS, 1.92%), rs200505918 (not reported, 1.92%), and rs147633212 (VUS, 1.92%). Under “Variants in DNA Damage Repair Genes”, the DNA polymerase gene *POLK* was the gene with most frequent germline variants: rs148960463 (Pathogenic, 3.85%), rs368533237 (not reported, 1.92%), rs151251843 (not reported, 1.92%), g.chr5:75596636A>G (not reported, 1.92%), and g.chr5:75581377C>G (not reported, 1.92%).

Under “Common Variants”, the top 5 most frequent single nucleotide polymorphisms (SNPs), previously shown in genome wide association studies (GWASes) to be associated with glioma, were: rs1110784 (in *ATP9B*, 65.38%), rs1760897 (in *TEP1*, 48.08%), rs3828550 (in *KDR*, 11.54%), rs1799782 (in *XRCC1*, 7.69%) and rs1468358 (in *PLOD3*, 3.85%).

#### Somatic short variants

The top 10 genes that harbored a reported somatic short variant per each subsection are presented in Fig. 4b. Overall, there was a positive correlation between the percentages of all the top reported genes between the current cohort (NOT) and the TCGA cohort (Spearman’s *ρ* = 0.5, *p* < 0.001). Except for the subsection “Variants in DNA Damage Repair Genes", there were positive correlations of percentages of the top 10 genes between the current cohort (NOT) and the TCGA cohort per each subsection (Fig. 4b, top figure). It can be observed that the most frequent genes were observed in “Variants in Established Glioma Genes”. The gene with the most frequent somatic short variants was *TP53* (41.38%).

Figure 4b bottom figure displays the distributions of VAFs of top 10 genes with reported somatic short variants per each somatic short variant subsection. The distributions of tumor VAF values per subsection were significantly different (Kruskal–Wallis test, *p* < 0.001). The median VAF of “Variants in Established Glioma Genes” was the highest (0.38), followed by “Hotpot Variants in Cancer Gene Census Genes” (0.31), “Other Variants in Cancer Gene Census Genes” (0.28), “Variants in Important KEGG Pathway Genes” (0.22) and “Variants in DNA Damage Repair Genes” (0.19).

#### Somatic copy number alterations

Under “Established SCNAs in Glioma”, the most frequently reported SCNA events were *CDK6* amplification (55.17% of analyses), *MET* amplification (50%), *CDKN2A* deletion (44.83%), *EZH2* amplification (44.83%), *BRAF* amplification (43.1%), *CDKN2B* deletion (43.1%), *EGFR* amplification (39.66%), *RB1* deletion (22.41%), *MAX* deletion (20.69%), and *NF2* deletion (20.69%, Fig. 4c, left).

Chromosomal-arm-level SCNA events in each tumor are presented in Additional file 2: Fig. S2. The most frequently observed chromosome or chromosomal arm level SCNAs were chr7 amplification (46.55%), chr22q loss (34.48%), chr10 loss (27.59%), chr10q loss (25.86%), chr13q loss (24.14%), chr9p loss (22.41%), chr14q loss (18.97%), chr20q amplification (15.52%), chr21p loss (15.52%), and chr6q loss (15.52%, Fig. 4C, right). Frequencies of all SCNA events by cytoband are presented in Additional file 2: Fig. S4, amplifications in chr7 and deletions in chr10 have the highest overall frequencies.

### Personalized neurooncology report

**Fig. 5:**
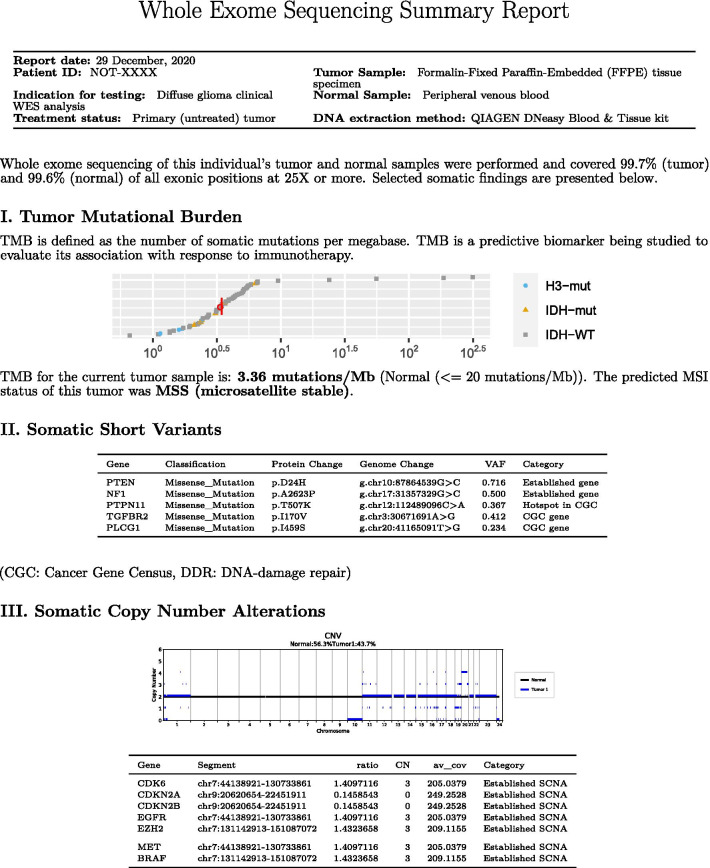
Example whole exome sequencing summary report

To communicate the findings of potential clinical relevance, we developed a comprehensive personalized neurooncology report (Fig. 5, Additional file 1).

#### Summary report of somatic WES findings

The initial page of the report summarizes somatic findings, including TMB, somatic short variants, and somatic copy number alterations on a single page (Fig. 5). The summary includes a description section, providing information on the indication for testing, treatment status, tumor sample type, normal sample type, and DNA extraction method. The summary indicates exome coverage, providing a high-level overview of the quality of the patient's exome data.

For TMB, a plot of all previously analyzed tumor samples’ TMB values, along with the current tumor’s TMB (circled in red) by molecular subset (devised based on WES-identifiable markers) is provided, and the TMB of the current is indicated. The MSI status of the tumor is also indicated in this section.

For somatic short variants, all somatic short variants reported in different categories (i.e., “Variants in Established Glioma Genes”, “Hotspot Variants in Cancer Gene Census Genes”, “Other Variants in Cancer Gene Census Genes”, “Variants in DNA Damage Repair Genes” and “Variants in Important KEGG Pathway Genes”) are presented in a table format, containing information on variant impact classification, protein change annotation, genome change annotation, and tumor VAF.

For SCNAs, a plot of copy number of segments by chromosome is provided as well as a table containing all gene-level SCNAs reported in each category (i.e. “Established SCNAs in Glioma” and “SCNAs in Cancer Gene Census Genes”.

#### Quality metrics

(I) **Summary table of quality metrics**
  A summary of sequencing quality, including the number of lanes, read type, read length, the total number of reads, PF reads, aligned PF reads, PE aligned, mean coverage, and percentage of bases covered at 1X, 5X, 10X, 25X, 50X and 100X, is reported here.
(II) **Tumor purity**

The fraction of reads coming from cross-sample contamination, reflecting a measure of tumor purity, is calculated using the GATK-CalculateContamination tool and presented here. A purity/clonality estimate (reflecting normal contamination in the tumor sample) based on copy number alterations is presented under section “Tumor Heterogeneity Analysis”.

#### Germline alterations

Findings are filtered (except for “Common Variants”) for germline single nucleotide variations (SNVs) and short (typically less than 20 bases-long) insertion-deletion events (indels) that:

- are not reported as "benign" or "likely benign" in ClinVar [25]
- have population Minor Allele Frequency (MAF) < 1% (in 1000 Genomes [27], ExAC [28] and ESP6500[29])
- have non-synonymous impact (one of "Frame_Shift_Del", "Frame_Shift_Ins", "Splice_Site", "Translation_Start_Site","Nonsense_Mutation", "Nonstop_Mutation", "In_Frame_Del","In_Frame_Ins", "Missense_Mutation")
- are not in genes that are often non-pathogenic and passengers but are frequently mutated in most of the public exome studies (named FLAGS) as collected by Shyr et al. [26]

The section follows a sequential order (except for “Common Variants”) where an alteration reported in a subsection is not reported in the following subsections.

- ACMG incidental findings
  Filtered germline SNV/indels affecting any ACMG SF v2.0 [30] genes for reporting incidental findings are reported here.
- Variants in cancer gene census genes
  This subsection filters the germline SNV/indels for genes that are in the Cancer Gene Census (CGC) from the Catalogue of Somatic Mutations in Cancer (COSMIC), a catalog of genes containing variants associated with cancer [31].
- Variants in cancer predisposition genes
  Genes in which germline variants confer an increased risk of cancer are called cancer predisposition genes. Filtered germline SNV/indels in cancer predisposition genes cataloged by Rahman [32] are reported here.
- Variants in DNA damage repair genes
  Germline variants in genes that take part in the DNA damage repair as collected by the Wood laboratory[33] are reported here.
- Common variants

Here, germline alterations are filtered for single nucleotide polymorphisms (SNPs) previously shown in genome-wide association studies (GWASes) to have an association with gliomas, as listed in the GWAS catalog [34] under “EFO_0005543” [35].

#### Somatic SNV/indels

Somatic variants obtained via MuTect2 are filtered to have a variant allele frequency (VAF) of at least 5% and be non-synonymous variants. FLAGS [26] were excluded from the report. The variant subsections in this section also follow a sequential order.

- Tumor mutational burden
  In this subsection, the Tumor Mutational Burden (TMB) is reported. TMB is defined as the number of somatic mutations in the coding region per megabase, including SNVs and indels. This calculation is performed through:
  1. keeping variants with VAF > 5% and.
  2. keeping variants with a sequence depth > 20X in the tumor and > 10X in the normal.
  Two scatter plots and a table summarize the median TMB values overall and for each molecular subset (devised based on WES-identifiable markers) for the current and all previously reported tumors.
- Microsatellite instability status
  The MSI status is predicted using the tool MSIpred [36]. Additionally, polymerase-epsilon deficiency is predicted based on the presence of (a) somatic SNVs/Mb > 60 and (b) somatic indels in single sequence repeats/Mb < 0.18.
  The predicted MSI and polymerase-epsilon deficiency statuses are reported here.
- Variants in established glioma genes
  This subsection contains somatic SNV/indels in genes reported in the TCGA pan-glioma study of Ceccarelli et al., which analyzed 1122 WHO grade II-III and IV diffuse-gliomas [15].
- Hotspot variants in cancer gene census genes
  This subsection presents somatic SNV/indels where (a) the gene harboring the variant is listed in CGC and (b) the variant is observed in multiple tumors in COSMIC.
- Other variants in cancer gene census genes
  This subsection lists other somatic variants where the gene harboring the variant is listed in CGC (and the variant is not a hotspot variant).
- Other possibly important somatic SNV/indels
- Variants in DNA damage repair genes
  Contains possibly important somatic SNV/indels in DNA damage repair genes that are in the list of Human DNA Repair Genes[33]
- Variants in important KEGG pathway genes

Contains possibly important somatic SNV/indels in selected KEGG [37] pathways (namely “Cell cycle”, “mTOR signaling” and “Pathways in cancer”).

#### Somatic copy number alterations (SCNAs)

For high confidence, only SCNAs with a |log_2_(Tumor/Normal) ratio|≥ 0.25 are reported.

- SCNA Burden
  Numerous studies have shown that SCNA burden is an important prognostic marker [38–41]. In this subsection, 4 metrics of SCNA burden are reported:
- Total altered length(Mb)
- Total number of alterations
- The average length of alterations(kb) = Total altered length/Total number of alterations
- Weighted Genome Instability Index = estimate of the proportion of the exome with aberrant copy number, weighted on a per chromosome basis
- Established SCNAs in Glioma
  This subsection presents SCNAs that are in a list of gene-level SCNAs curated because of their importance in gliomas (as reported in the aforementioned TCGA pan-glioma study [15]).
- SCNAs in Cancer Gene Census Genes
  This subsection lists SCNAs where the gene subject to copy-number alteration is listed in CGC.
- Broad SCNAs
  This subsection lists SCNA events that span over one or more cytobands.
- Plots of SCNA Segments by Chromosome

This subsection displays SCNA plots (log_2_(Tumor/Normal) ratio vs. position) per all chromosomes.

#### Loss of Heterozygosity (LOH) events

For high confidence, only LOH events for which the absolute difference of B-allele frequencies (|BAF_Tumor_ − BAF_Normal_|) is larger than 0.4 are reported.

- LOH overview
  All LOH events that pass the filter are reported here.
- LOH + somatic SNV/indel
  Here, alterations where a gene has LOH, and a somatic SNV/indel are reported.
- LOH events in CGC genes

LOH events where the gene subject to LOH is listed in CGC are reported here.

#### Genes with double hit

A double hit strongly suggests a relevant tumor suppressor gene [42]. In this section, the list of genes with somatic SNV/indel as well as SCNA and/or LOH events are reported.

#### Tumor heterogeneity analysis

To estimate tumor purity as well as clonal/subclonal SCNAs, THetA [43] is used. In this section, the results of THetA are presented.

#### Mutational signatures

Somatic mutations in cancer genomes are caused by multiple mutational processes, each of which generates a characteristic mutational signature [14]. Analysis of mutational signatures is becoming routine in clinical cancer genomics as the detected signatures of mutational processes have implications for pathogenesis, classification, prognosis, and even treatment decisions [44, 45].

Using DeConstructSigs [46], the weights of Mutational Signatures v3 (May 2019) from COSMIC [47] are estimated. This section presents the mutational signatures detected in this tumor.

#### pathfindR—KEGG pathway enrichment analysis

For studying mechanisms underlying oncological processes, KEGG pathway enrichment analyses are performed using the active-subnetwork-oriented enrichment approach of pathfindR [48].

- Enrichment Results for High-impact Somatic SNV/indels
  Genes harboring any somatic non-synonymous variants with a VAF > 5% and not in FLAGS are used for analysis.
- Enrichment Results for High-impact SCNA

Genes harboring homozygous deletion(Tumor/Normal ratio < 0.5) or multi-copy amplification(Tumor/Normal ratio > 1.5) are used for analysis.

## Discussion

The reporting of findings of potential oncological relevance from NGS is rapidly expanding into the clinical area [1–3]. In this work, we aimed to present the efficiency and utility of our approach to analyze whole-exome sequencing data of individual gliomas and produce clinically interpretable reports of individual cancer genomes. The approach attempts sequential filtration of various layers of genetic information to assist in clinical decision-making.

It is established that individual tumors may harbor clinically relevant alterations which are not observed frequently in tumors of the same cancer type [49]. In our approach, alterations are prioritized from “highly likely” to “less likely” to be clinically relevant. This is done by sequentially filtering for (1) glioma-related alterations followed by (2) cancer-related alterations followed by (3) alterations in selected gene sets. Through sequential filtering, NOTATES greatly reduces the number of variants to be reported while still retaining the most clinically relevant variants as well as other variants of potential significance.

The clinical interpretation of germline variants in cancer is challenging. The sequential reporting of germline variants in NOTATES allows the clinician to identify any clinically relevant variants. The “ACMG Incidental Findings” section allows the identification of incidental variants, followed by “Variants in Cancer Gene Census Genes” and “Variants in Cancer Predisposition Genes” allowing the identification of cancer-related variants. “Variants in DNA Damage Repair Genes” specifically lists germline variants in DNA damage repair genes, which are important in gliomas because numerous studies have provided evidence that DNA repair deficiency was a central theme in gliomagenesis, a finding also reported in our previous study [50, 51]. Most reported germline variants were not included in ClinVar. As previously reported, the prevalence of “pathogenic” / “likely-pathogenic” germline variants in the ACMG Secondary Findings v2.0 list was low [52] whereas the prevalence of such variants in the cancer-related subsections were relatively higher (among variants with clinical significance annotation in ClinVar).

For somatic SNV/indels, the subsection “Variants in Established Glioma Genes” contains the most likely glioma-specific drivers. Overall, a third of the somatic SNVs reported were in this subsection per tumor. The two following subsections contain somatic variants in CGC genes, pointing to possible oncogenic alterations that are not tumor-type-specific. Hotspot alterations were infrequent but a third of the reported variants per tumor were alterations in CGC genes. The median VAF of the glioma-specific alterations (reported under “Variants in Established Glioma Genes”) was relatively higher than that of alterations reported in the other subsections, emphasizing the importance of this subsection.

For assessing SCNAs, both broad (cytoband-level) and gene-level SCNA events are reported. The most commonly observed (observed in > 25%) chromosomal or arm-level copy-number alterations were chr7 amplification, chr22q deletion, and chr10 deletion, frequently observed alterations in gliomas [53–55]. When filtered for SCNAs reported in the TCGA-pan glioma study (presented under “Established SCNAs in Glioma”), each tumor contained a median of 7 such gene-level SCNAs. The most common (observed in > 25%) such SCNA events were CDK6 amplification, MET amplification, BRAF amplification, EZH2 amplification, PTEN deletion, CDKN2A deletion, CDKN2B deletion, EGFR amplification.

TMB and the predicted MSI status, which are both predictive biomarkers for systemic cancer immunotherapy [56–58], are included in the report as well. Rather than only providing a hard cut-off value, we provide a plot and a table summarizing the TMB status of all reported gliomas, which enables the clinician to evaluate the TMB status in the relevant context. The TMB distribution of this glioma cohort was similar to those of the TCGA glioma cohorts. As expected, the median TMB value for recurrent tumors was higher than the primary tumors. The TMB values of different glioma molecular subsets were also different. Along with TMB, we also predict MSI status and possible POLE deficiency. As previously reported, the incidence of MSI in diffuse gliomas was low[59–61].

Because NOTATES allows the identification of specific genetic alterations indicating differing clinical outcomes in gliomas, the findings in the NOTATES report reflect the severity of the tumor. For example, if a mutation in IDH1/IDH2 is detected, this indicates a better prognosis [62, 63], whereas H3-K27M or G34 mutations imply worse disease outcome [64, 65]. Similarly, IDH-wild-type gliomas with EGFR amplifications and/or chromosome 7 amplifications and chromosome 10 loss can be molecularly defined as GBM, conferring worse prognosis [66, 67]. In addition to specific genetic alterations, NOTATES calculates TMB and evaluates the presence of MSI, further aiding the clinical assessment because these are both predictive biomarkers for systemic cancer immunotherapy [56–58].

It is important to emphasize that all findings presented in the NOTATES report complement each other. For example, a high TMB, predicted MSI, somatic variants in mismatch repair genes and mismatch repair deficiency-related mutational signatures will all support highly likely mismatch repair deficiency in a tumor, indicating a higher chance of response to immunotherapy.

Identification of clinically relevant findings from the vast amount of data produced by WES is a substantial challenge [49, 68]. In this work, we aimed to propose a solution to this issue by presenting our approach for reporting of genomic findings from WES data of individual gliomas. Using curated resources, NOTATES investigates and presents various forms of findings of potential clinical importance: germline short variants, somatic short variants, somatic copy-number alterations, loss-of-heterozygosity events, tumor mutational burden, microsatellite instability, and mutational signatures. The NOTATES report is formatted to provide a coherent overview of clinically-relevant genomic findings, enabling the adaptation of WES to the clinical setting. For this purpose, NOTATES utilizes curated sets of relevant genes and databases that collect knowledge about cancer alterations and their relationships to tumor formation and clinical utility and reports the findings in a sequential manner according to clinical relevance. The results in this work demonstrate that NOTATES successfully captures glioma-specific alterations while also reporting possibly relevant cancer-related alterations. The comprehensive report contains the most clinically important findings that may aid in clinical decision-making.

## Conclusions

In this work, we presented the outline of and a compilation of results from our WES analysis workflow. The results display that NOTATES produces clinically relevant results in a concise but exhaustive manner. Through this work, we demonstrate that WES can practically and efficiently be adapted to the clinical setting for the analysis of individual gliomas.

## Supplementary Information

**Additional file 1.** An Example Personalized Neurooncology Report.PDF file containing an example report for a diffuse glioma tumor sample.

**Additional file 2.** Supplementary figures and tables.

## Data Availability

The datasets used and/or analyzed during the current study are available from the corresponding author on reasonable request. NOTATES is available for non-commercial purposes.

## Abbreviations

NGS: Next generation sequencing
WES: Whole exome sequencing
WGS: Whole genome sequencing
FFPE: Formalin-fixed paraffin-embedded
SNV: Single nucleotide variant
indel: Insertion/deletion
SCNA: Somatic copy number alteration
MSI: Microsatellite instability
TMB: Tumor mutational burden
LOH: Loss of heterozygosity
VAF: Variant allele frequency
VUS: Variant of unknown significance
SD: Standard deviation
MAF: Minor allele frequency
CGC: Cancer gene census
COSMIC: Catalogue of somatic mutations in cancer
SNP: Single nucleotide polymorphism
GWAS: Genome-wide Association Study
LGG: Lower-grade glioma
GBM: Glioblastoma multiforme
