# Supplementary material for: Sequential filtering for clinically relevant variants as a method for clinical interpretation of whole exome sequencing findings in glioma
Source: BMC Med Genomics. 2021 Feb 23;14:54. doi: 10.1186/s12920-021-00904-3 (PMC7903763; doi:10.1186/s12920-021-00904-3)
Supplement: Supplementary file 1 — Additional file 1. An Example Personalized Neurooncology Report.PDF file containing an example report for a diffuse glioma tumor sample. [file 12920_2021_904_MOESM1_ESM.pdf]

Whole Exome Sequencing Summary Report

Report date: 29 December, 2020
Patient ID: NOT-XXXX
Indication for testing: Diffuse glioma clinical WES analysis
Treatment status: Primary (untreated) tumor
Tumor Sample: Formalin-Fixed Paraffin-Embedded (FFPE) tissue specimen
Normal Sample: Peripheral venous blood
DNA extraction method: QIAGEN DNeasy Blood & Tissue kit

Whole exome sequencing of this individual's tumor and normal samples were performed and covered 99.7% (tumor) and 99.6% (normal) of all exonic positions at 25X or more. Selected somatic findings are presented below.

I. Tumor Mutational Burden

TMB is defined as the number of somatic mutations per megabase. TMB is a predictive biomarker being studied to evaluate its association with response to immunotherapy.

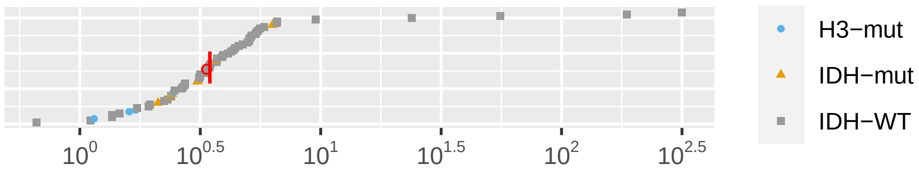

TMB for the current tumor sample is: **3.36 mutations/Mb** (Normal (<= 20 mutations/Mb)). The predicted MSI status of this tumor was **MSS (microsatellite stable)**.

II. Somatic Short Variants

Gene	Classification	Protein Change	Genome Change	VAF	Category
PTEN	Missense_Mutation	p.D24H	g.chr10:87864539G>C	0.716	Established gene
NF1	Missense_Mutation	p.A2623P	g.chr17:31357329G>C	0.500	Established gene
PTPN11	Missense_Mutation	p.T507K	g.chr12:112489096C>A	0.367	Hotspot in CGC
TGFBR2	Missense_Mutation	p.I170V	g.chr3:30671691A>G	0.412	CGC gene
PLCG1	Missense_Mutation	p.I459S	g.chr20:41165091T>G	0.234	CGC gene

(CGC: Cancer Gene Census, DDR: DNA-damage repair)

III. Somatic Copy Number Alterations

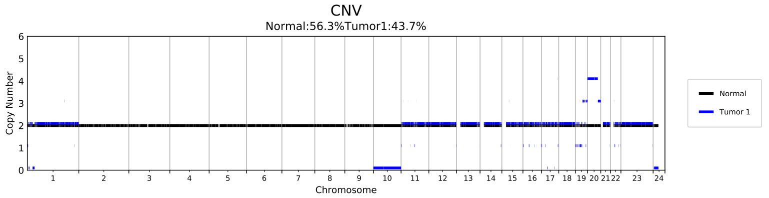

Gene	Segment	ratio	CN	av_cov	Category
CDK6	chr7:44138921-130733861	1.4097116	3	205.0379	Established SCNA
CDKN2A	chr9:20620654-22451911	0.1458543	0	249.2528	Established SCNA
CDKN2B	chr9:20620654-22451911	0.1458543	0	249.2528	Established SCNA
EGFR	chr7:44138921-130733861	1.4097116	3	205.0379	Established SCNA
EZH2	chr7:131142913-151087072	1.4323658	3	209.1155	Established SCNA
MET	chr7:44138921-130733861	1.4097116	3	205.0379	Established SCNA
BRAF	chr7:131142913-151087072	1.4323658	3	209.1155	Established SCNA

Neurooncological Whole Exome Sequencing Report

NOT ID: NOT-XXXX
Treatment status: Primary (untreated) tumor
Tumor sample: Formalin-Fixed Paraffin-Embedded (FFPE) tissue specimen
Normal sample: Peripheral venous blood
DNA extraction method: QIAGEN DNeasy Blood & Tissue kit
Report date: 29 December, 2020
The reference genome assembly ID: hg38

I. Quality Metrics

Ia. Summary Table of Quality Metrics

	Normal	Tumor
Number of Lanes	1	1
Read Type	Paired-End	Paired-End
Read Length	101	101
Total Number of Reads(in millions)	172.80	298.23
PF Reads*	100%	100%
Aligned PF Reads**	99.89%	99.9%
PE Aligned***	99.91%	99.93%
Mean Coverage	183.74	273.95
1X	99.9%	99.9%
5X	99.9%	99.9%
10X	99.8%	99.8%
25X	99.6%	99.7%
50X	98.3%	99.1%
100X	87.1%	95.4%

* PF is defined as passing Illumina's filter
** The percentage of PF reads that aligned to the reference sequence
*** The percentage of reads whose mate pair was also aligned to the reference

Ib. Tumor Purity

The fraction of reads coming from cross-sample contamination, reflecting a measure of tumor purity, is calculated using the GATK - `CalculateContamination` tool. This tool calculates the fraction of reads coming from cross-sample contamination and estimates contamination based on the signal from reference reads at homozygous alternate sites. A purity/clonality estimate (reflecting normal contamination in the tumor sample) based on copy number alterations (CNAs) is presented under section VII. Tumor Heterogeneity Analysis.

contamination	error
0.0003895	6.45e-05

II. Germline Alterations

Germline alterations are assessed from the WES sequencing of DNA extracted from blood. Alterations are called using GATK - HaplotypeCaller. Findings are filtered for germline single nucleotide variations (SNVs) and short (typically less than 20 bases-long) insertion-deletion events (indels) that:

- have a MAF $< 1\%$ in gnomAD, ExAC, ESP-6500 and 1000 Genomes projects
- have been not reported as "benign" or "likely benign" in ClinVar
- have non-synonymous impact
- are not in genes that are often non-pathogenic and passengers but are frequently mutated in most of the public exome studies (named FLAGS) as collected by Shyr et al. [1].

(except for the subsection "Common Variants"). The altered gene, the position of the SNV, the reference nucleotide as well as the altered nucleotide, the mutation type, the read depth of the variation, the estimated allelic frequency and clinical significance (as reported in ClinVar) are reported. The report follows a sequential order where an alteration reported in a subsection is not reported in the following subsections. This sequential order prioritizes variants from a "highly likely" to "less likely" clinical relevance.

II. a. ACMG Incidental Findings

Filtered germline SNV/indels that affects any of the genes listed in ACMG SF V2.0[2] - incidental findings are reported under this subsection.

Gene	rs_id	Chr	Pos	Disease.s.	Effect	Ref	Alt	Ref_depth	Alt_depth	AF	Significance
CACNA1S	https://www.ncbi.nlm.nih.gov/snp/rs1224786667	chr1	201049077	Malignant hyperthermia susceptibility	Missense_Mutation	C	T	48	62	0.5	not reported
TP53		chr17	7676154	glioma predisposition gene, Li-Fraumeni Syndrome, Other conserved DNA damage response genes	Missense_Mutation	G	C	0	256	NA	drug_response
APOB	https://www.ncbi.nlm.nih.gov/snp/rs1339117465	chr2	21002271	Familial hypercholesterolemia	Missense_Mutation	A	G	86	66	0.5	Pathogenic

II. b. Variants in Cancer Gene Census Genes

Cancer Gene Census (CGC) from the Catalogue of Somatic Mutations in Cancer (COSMIC) database is a catalog of genes which contain mutations that have been causally linked to cancer (https://cancer.sanger.ac.uk/census). This subsection filters the germline SNV/indels for genes that are reported in CGC.

Gene	rs_id	Chr	Pos	Effect	Ref	Alt	Ref_depth	Alt_depth	AF	Significance
ATM		chr11	108345895	Missense_Mutation	T	A	61	76	0.5	not reported
ATM	https://www.ncbi.nlm.nih.gov/snp/rs749193688	chr11	108345896	Missense_Mutation	A	G	61	77	0.5	VUS
ATM		chr11	108345899	Missense_Mutation	T	A	61	77	0.5	not reported
PRPF40B	https://www.ncbi.nlm.nih.gov/snp/rs752705577	chr12	49643933	Missense_Mutation	G	A	119	122	0.5	not reported
SH2B3	https://www.ncbi.nlm.nih.gov/snp/rs751562450	chr12	111418569	Missense_Mutation	C	T	75	57	0.5	not reported
FLT3		chr13	28050157	Missense_Mutation	G	A	0	149	NA	not reported
USP6		chr17	5139503	Missense_Mutation	C	T	253	262	0.5	not reported
SETBP1		chr18	44950895	Missense_Mutation	C	A	99	85	0.5	not reported
EML4	https://www.ncbi.nlm.nih.gov/snp/rs113909364	chr2	42245532	Missense_Mutation	C	T	55	57	0.5	not reported
EPAS1	https://www.ncbi.nlm.nih.gov/snp/rs147672406	chr2	46382550	Missense_Mutation	G	A	121	107	0.5	not reported
MN1		chr22	27799795	Missense_Mutation	G	C	139	158	0.5	not reported
PTPN13	https://www.ncbi.nlm.nih.gov/snp/rs760147391	chr4	86799171	Missense_Mutation	A	G	59	38	0.5	not reported
IL2	https://www.ncbi.nlm.nih.gov/snp/rs1310812139	chr4	122456387	Missense_Mutation	T	C	52	56	0.5	not reported
CDKN1A	https://www.ncbi.nlm.nih.gov/snp/rs752557277	chr6	36684126	Missense_Mutation	C	T	74	68	0.5	not reported
PABPC1	https://www.ncbi.nlm.nih.gov/snp/rs1181241722	chr8	100706996	Splice_Site	-	GGATGAGGTCTGGCACCCTGAGCAGTCCAGCGAG	113	25	0.5	not reported
PSIP1		chr9	15479669	Missense_Mutation	T	C	52	67	0.5	not reported
FLNA		chrX	154359839	Missense_Mutation	G	A	0	94	NA	Conflicting

II. c. Variants in Cancer Predisposition Genes

Genes in which germline mutations confer highly or moderately increased risks of cancer are called cancer predisposition genes. Filtered germline SNV/indels cataloged in the 2014 Nature publication by Rahman [3], studying cancer predisposition genes, are reported in this subsection.

[1] Shyr C, Tarailo-graovac M, Gottlieb M, Lee JJ, Van karnebeek C, Wasserman WW. FLAGS, frequently mutated genes in public exomes. BMC Med Genomics. 2014;7:64.

[2] Kalia SS, Adelman K, Bale SJ, et al. Recommendations for reporting of secondary findings in clinical exome and genome sequencing, 2016 update (ACMG SF v2.0): a policy statement of the American College of Medical Genetics and Genomics. Genet Med. 2017;19(2):249-255.

[3] Rahman N. Realizing the promise of cancer predisposition genes. Nature. 2014;505(7483):302-8.

Gene	rs_id	Chr	Pos	Effect	Ref	Alt	Ref_depth	Alt_depth	AF	Significance
DOCK8	https://www.ncbi.nlm.nih.gov/snp/rs374952856	chr9	334277	Missense_Mutation	G	A	86	98	0.5	not reported

II. d. Variants in DNA Damage Repair Genes

In this subsection, filtered germline variants in Human DNA Repair Genes [4] are presented.

Gene	rs_id	Chr	Pos	Function	Effect	Ref	Alt	Ref_depth	Alt_depth	AF	Significance
EME2		chr16	1776227	Homologous recombination	Missense_Mutation	C	G	67	74	0.5	not reported
EME1	https://www.ncbi.nlm.nih.gov/snp/rs141978919	chr17	50375548	Homologous recombination	Missense_Mutation	G	A	104	103	0.5	not reported
FAAP100	https://www.ncbi.nlm.nih.gov/snp/rs1211638985	chr17	81550962	Fanconi anemia	In_Frame_Del	AGG	-	153	161	0.5	not reported
XAB2	https://www.ncbi.nlm.nih.gov/snp/rs1304347185	chr19	7620313	NER-related	Missense_Mutation	G	A	123	120	0.5	not reported
PAXIP1	https://www.ncbi.nlm.nih.gov/snp/rs772247568	chr7	154969059	Homologous recombination	Missense_Mutation	T	C	78	74	0.5	not reported
SPIDR		chr8	47673841	Homologous recombination	Missense_Mutation	G	A	94	82	0.5	not reported
RAD54B	https://www.ncbi.nlm.nih.gov/snp/rs114436457	chr8	94387141	Homologous recombination	Missense_Mutation	T	G	73	57	0.5	not reported

II. e. Common Variants

This subsection is distinct from the previous germline alterations subsections. Here, germline alterations are filtered for “single nucleotide polymorphisms (SNPs)” which were previously shown in genome wide association studies (GWAS) to have an association with gliomas and were listed in the GWAS catalog (https://www.ebi.ac.uk/gwas/efotraits/EFO_0005543)

rs_id	Gene	Effect	Risk.Allele	Ref	Alt	Ref_depth	Alt_depth	AF
rs1760897	TEP1	Missense_Mutation	NA	A	G	0	288	1.0
rs1110784	ATP9B	Intron	NA	A	G	0	113	1.0
rs1799782	XRCC1	Nonsense_Mutation	TRUE	G	A	89	71	0.5

[4] Mdanderson.org. 2020. Human DNA Repair Genes. [online] Available at: https://www.mdanderson.org/documents/Labs/Wood-Laboratory/human-dna-repair-genes.html.

III. Somatic Single Nucleotide Variations (SNVs) and Small Insertion/Deletions (Indels)

In this section, Tumor Mutational Burden, Microsatellite Instability Status, and somatic mutations are reported.

Somatic mutations were called from the tumor-blood pair in the current analysis using GATK - MuTect2 with a cutoff of 5% variant allele frequency (VAF). Only non-synonymous variants (with variant classifications of High/Moderate variant consequences: "Frame_Shift_Del", "Frame_Shift_Ins", "Splice_Site", "Translation_Start_Site","Nonsense_Mutation", "Nonstop_Mutation", "In_Frame_Del","In_Frame_Ins", "Missense_Mutation", see http://www.ensembl.org/Help/Glossary?id=535) were used for reporting. Genes that are often non-pathogenic and passengers but are frequently mutated in most of the public exome studies (named FLAGS) as collected by Shyr et al. were excluded from the report.

The reporting of SNVs/indels follows a sequential order where an alteration reported in a subsection is not reported in the following subsections. This sequential order prioritizes variants from a "highly likely" to "less likely" clinical relevance.

III. a. Tumor Mutational Burden (TMB)

TMB is defined as the number of somatic mutations in the coding region (more accurately the exome capture kit's intervals) per megabase, including SNVs and indels.

This calculation was performed using the following filtering approach:

1. keeping variants with mutated allele frequency greater than 5%
2. keeping variants with a sequence depth greater than 20X in the tumor sample greater and 10X in the normal sample

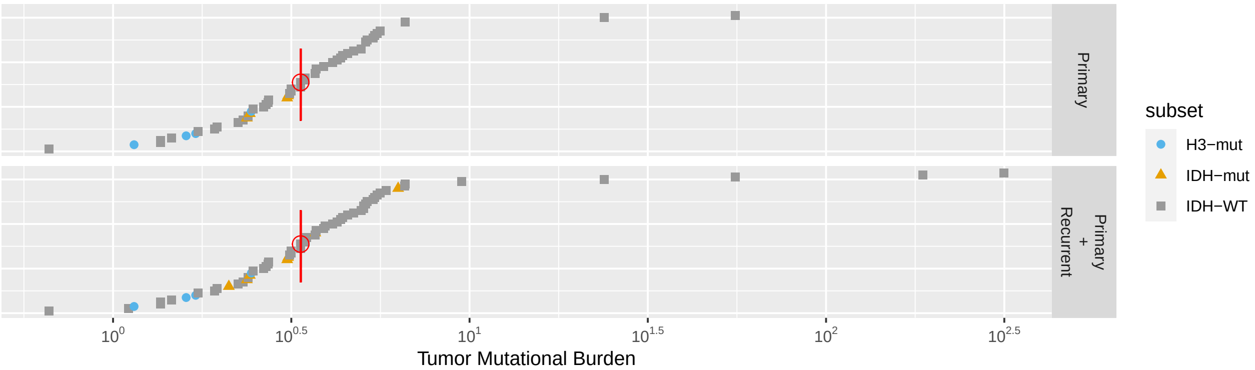

TMBs (mutations/Mb) of all tumors reported until now, including the current tumor, circled in red are presented in the above scatter plots. The plots separately displayed for (a) primary (untreated tumors only, top panel, n =50) and (b) primary + recurrent tumors (untreated tumors and tumors previously undergone treatment, bottom panel, n=63). The red vertical lines indicate median TMB for each panel. The table below displays the median TMBs per glioma molecular subsets over all previous analyses for primary and primary+recurrent tumors separately.

	Primary + Recurrent	Primary
H3-mut	1.654391	1.654391
IDH-mut	3.079712	2.749423
IDH-WT	3.703290	3.410590
Overall	3.461495	3.156068

TMB for the current tumor sample is: 3.36 mutations/Mb (Normal (<= 20 mutations/Mb))

III. b. Microsatellite Instability Status

Microsatellite instability (MSI) is the result of impaired DNA mismatch repair and constitutes a cellular phenotype of clinical significance in many cancer types. The MSI status of the tumor was predicted using the tool MSIpred [5]. MSIpred computes 22 somatic mutational features (predictors of MSI) and using these features, it predicts tumor MSI status as microsatellite instability high (MSI-H) or microsatellite stable (MSS) using a machine learning method, support vector machine.

Additionally, polymerase-epsilon (POLE) deficiency is predicted based on the presence of both of:

1. total count of somatic SNVs / Mb > 60 AND
2. count of somatic indels in single sequence repeats / Mb < 0.18

Tumor	Predicted_MSI_Status	Likely_POLE_deficiency
NOT-0086	MSS	False

III. c. Variants in Established Glioma Genes

This subsection contains somatic SNV/indels in genes that have been reported in the TCGA pan-glioma study of Ceccarelli et al. [6] which analyzed 1122 WHO grade II-III and IV diffuse-gliomas.

Gene	Variant Classification	Protein	Genomic Change	VAF	COS_n_ov	UniProt_Region	Irino	TMZ	COSMIC
PTEN	Missense_Mutation	p.D24H	g.chr10:87864539G>C	0.716	13		no	no	https://cancer.sanger.ac.uk/cosmic/gene/analysis?ln=PTEN
NF1	Missense_Mutation	p.A2623P	g.chr17:31357329G>C	0.5	1		no	no	https://cancer.sanger.ac.uk/cosmic/gene/analysis?ln=NF1

III. d. Hotspot Variants in Cancer Gene Census Genes

This subsection presents somatic SNV/indels which fulfill the following criteria:

1. The gene harboring the variants is listed in the Catalogue of Somatic Mutations in Cancer (COSMIC) - CGC, which is a catalogue of genes which contain mutations that have been causally linked to cancer (https://cancer.sanger.ac.uk/census).
2. The variant has a mutation record in COSMIC at the same genomic position (as a recurrent mutation increases the likelihood of an actionable "gain-of function" mutation).

Gene	Variant Classification	Protein	Genomic Change	VAF	COS_n_ov	UniProt_Region	Irino	TMZ	COSMIC
PTPN11	Missense_Mutation	p.T507K	g.chr12:112489096C>A	0.367	1		no	no	https://cancer.sanger.ac.uk/cosmic/gene/analysis?ln=PTPN11

III. e. Other Variants in Cancer Gene Census Genes

This subsection lists somatic variants where the gene harboring the variants is listed in the COSMIC-CGC, which is a catalog of genes which contain mutations that have been causally linked to cancer (https://cancer.sanger.ac.uk/census).

Gene	Variant Classification	Protein	Genomic Change	VAF	COS_n_ov	UniProt_Region	Irino	TMZ	COSMIC
TGFBR2	Missense_Mutation	p.I170V	g.chr3:30671691A>G	0.412	0		no	no	https://cancer.sanger.ac.uk/cosmic/gene/analysis?ln=TGFBR2
PLCG1	Missense_Mutation	p.I459S	g.chr20:41165091T>G	0.234	0		no	no	https://cancer.sanger.ac.uk/cosmic/gene/analysis?ln=PLCG1

III. f. Other Possibly Important Somatic SNV/indels

Somatic SNV/indels in DNA damage repair genes that were presented on https://www.mdanderson.org/documents/Labs/Wood-Laboratory/human-dna-repair-genes.html:

[5] Wang C, Liang C. MSIpred: a python package for tumor microsatellite instability classification from tumor mutation annotation data using a support vector machine. Sci Rep. 2018;8(1):17546.

[6] Ceccarelli M, Barthel FP, Malta TM, et al. Molecular Profiling Reveals Biologically Discrete Subsets and Pathways of Progression in Diffuse Glioma. Cell. 2016;164(3):550-63.

```
No variation to report.
```

Somatic SNVs/indels in important KEGG Pathway genes:

```
No variation to report.
```

IV. Somatic Copy Number Alterations (SCNAs)

Only SCNAs with a $log_2(Tumor/Normal)$ ratio ≤ -0.25 or ≥ 0.25 were used in analysis.

IV. a. SCNA Burden

Numerous studies have shown that SCNA burden is an important prognostic marker[7,8,9]. Below are 4 metrics associated with SCNA Burden:

Total Altered Length (Mbp)	7.18
Weighted Genome Instability Index (%)	20.01
Total Number of Alterations	1360
Average Length of Alterations (kbp)	5.28

IV. b. Established SCNAs in Glioma

This is the intersection of SCNAs in this case with a list of SCNAs manually curated because of their importance in gliomas.

Gene	Segment	ratio	CN	av_cov
CDK6	chr7:44138921-130733861	1.4097116	3	205.0379
CDKN2A	chr9:20620654-22451911	0.1458543	0	249.2528
CDKN2B	chr9:20620654-22451911	0.1458543	0	249.2528
EGFR	chr7:44138921-130733861	1.4097116	3	205.0379
EZH2	chr7:131142913-151087072	1.4323658	3	209.1155
MET	chr7:44138921-130733861	1.4097116	3	205.0379
BRAF	chr7:131142913-151087072	1.4323658	3	209.1155

IV. c. SCNAs in Cancer Gene Census Genes

This subsection lists SCNAs where the gene subject to copy-number alteration is listed in CGC.

`No variation to report.`

IV. d. Broad SCNAs

This subsection lists SCNA events that span over one or more cytobands.

chromosome:start-end	cytoband	copy.number	ratio
chr1:6235717-10232434	chr1p36.23	1	0.5565951
chr1:25362461-34795215	chr1p35.3-chr1p35.1	1	0.5726796
chr4:85731-46979098	chr4p16.2-chr4p13	1	0.5680540
chr7:955293-18762277	chr7p22.2-chr7p21.2	3	1.3576391
chr7:18829461-44122459	chr7p15.3-chr7p14.1	3	1.4716147

[7] Hieronymus H, Murali R, Tin A, et al. Tumor copy number alteration burden is a pan-cancer prognostic factor associated with recurrence and death. Elife. 2018;7

[8] Hieronymus H, Schultz N, Gopalan A, et al. Copy number alteration burden predicts prostate cancer relapse. Proc Natl Acad Sci USA. 2014;111(30):11139-44.s

[9] Zhang L, Feizi N, Chi C, Hu P. Association Analysis of Somatic Copy Number Alteration Burden With Breast Cancer Survival. Front Genet. 2018;9:421.

(continued)

chromosome:start-end	cytoband	copy.number	ratio
chr7:44138921-130733861	chr7p12.3-chr7q32.1	3	1.4097116
chr7:131142913-151087072	chr7q33-chr7q35	3	1.4323658
chr7:151116793-159144771	chr7q36.2	3	1.4323658
chr10:47057-47474065	chr10p15.2-chr10q11.21	1	0.5922589
chr10:47995378-133626742	chr10q11.23-chr10q26.2	1	0.5826898
chr19:35270447-38394786	chr19q13.13	3	1.2850282
chr19:43962727-48365935	chr19q13.32	3	1.2810262
chr20:2755627-33439026	chr20p12.3-chr20q11.1	3	1.4510528
chr20:35004458-50151315	chr20q11.23-chr20q13.12	3	1.4546782
chr20:50275670-64273600	chr20q13.2-chr20q13.32	3	1.3160370
chrY:284188-2740848	chrYp11.31	1	0.5876382
chrY:5737272-25624550	chrYp11.1-chrYq11.223	0	0.1209179

IV. e. Plots of SCNA Segments by Chromosome

This subsection displays SCNA plots (log2(Tumor/Normal) ratio vs. position) per all chromosomes.

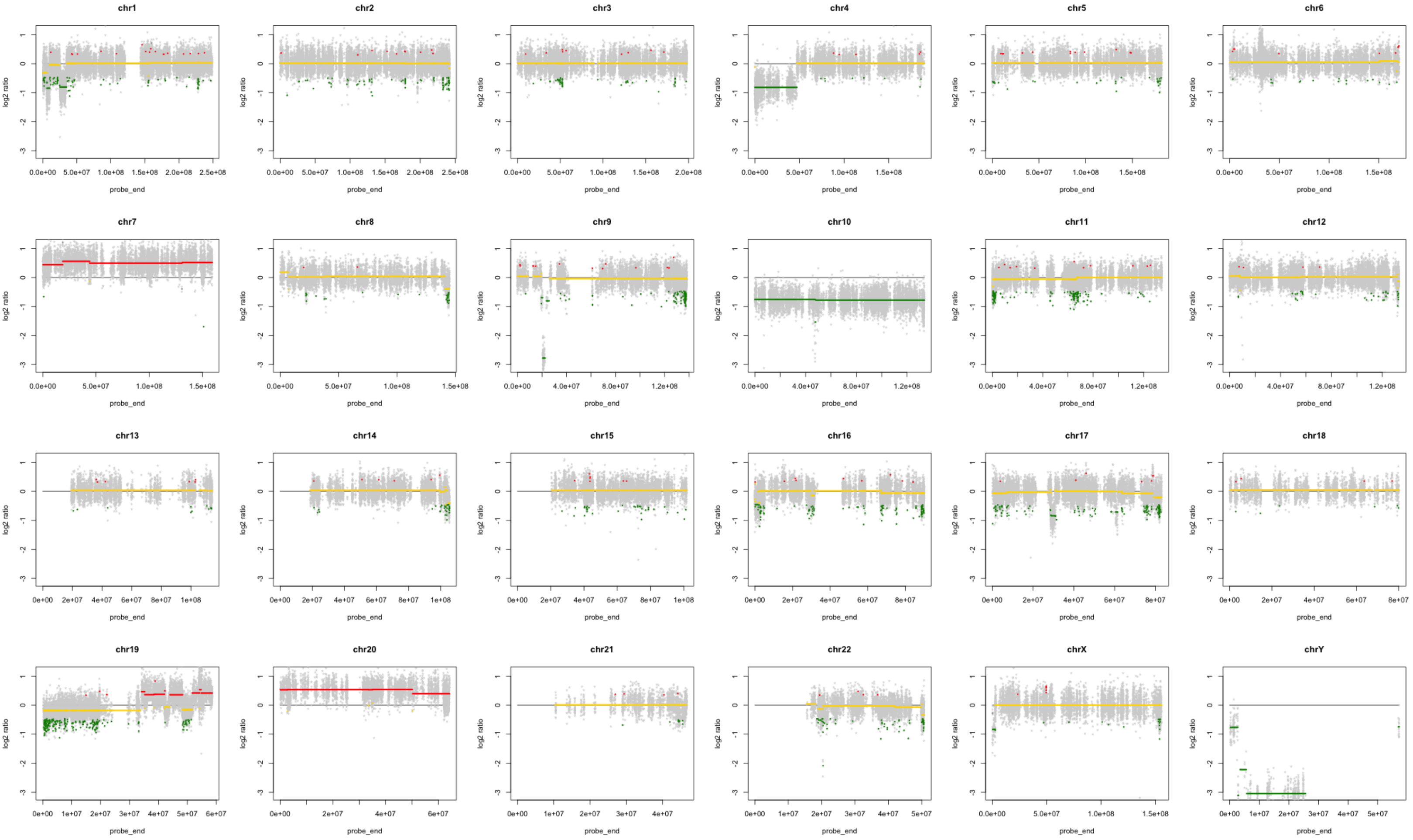

V. Loss of Heterozygosity (LOH) Events

Only LOH events for which absolute difference of B-allele frequencies ($|BAF_{Tumor} - BAF_{Normal}|$) is larger than 0.4 are reported.

V. a. LOH Overview

All LOH events that pass the filter are reported here.

Genes	Position	N_BAF	T_BAF	Absolute_Diff
PHF13, ZBTB48, TAS1R1, THAP3, KLHL21	chr1:6576401-6633042	0.4433781	0.8630807	0.4197026
RBP7	chr1:9997266-9997267	0.4347826	0.8591549	0.4243723
ZNF778	chr16:89233443-89233445	0.4362681	0.8507157	0.4144477
DEF8	chr16:89948839-89948854	0.2758621	0.7101449	0.4342829
>100	chr19:10001472-19634956	0.4831207	0.8910138	0.4078931
TMIGD2	chr19:4294626-4298388	0.5024752	0.9445471	0.4420719
UHRF1	chr19:4929401-4929461	0.4958848	0.9166667	0.4207819
UHRF1	chr19:4951052-4951053	0.4930556	0.9395161	0.4464606
UHRF1	chr19:4954443-4954444	0.4893617	0.9345550	0.4451933
KDM4B	chr19:5047680-5047681	0.4779874	0.9217877	0.4438003
RNF212	chr4:1056848-1056849	0.4388489	0.8602941	0.4214452
RNF212	chr4:1094113-1094114	0.3600000	0.8510638	0.4910638
MAEA, UVSSA	chr4:1309894-1355018	0.4605954	0.8609113	0.4003158
FBXL5	chr4:15655413-15655414	0.7230769	0.2000000	0.5230769
LDB2	chr4:16512125-16512126	0.3883495	0.7954545	0.4071050
TACC3, SLBP, TMEM129	chr4:1711859-1719030	0.4734895	0.8859813	0.4124918
TACC3	chr4:1731007-1740013	0.4653179	0.8679245	0.4026066
GAK	chr4:889057-889061	0.5000000	0.9057971	0.4057971
SLC2A9	chr4:9826475-9826476	0.4104478	0.8769231	0.4664753
SNORD100, RPS12	chr6:132816871-132816872	0.7619048	0.3333333	0.4285714

V. b. LOH + Somatic SNV/Indel

Here, alterations where a gene has LOH and a somatic SNV/indel are reported.

Gene	Segment	Absolute_Diff
SLC44A2	chr19:10001472-19634956	0.4078931

V. c. LOH Events in Cancer Gene Census Genes

LOH events where the gene subject to LOH is listed in CGC are reported here.

Gene	Segment	Absolute_Diff
DNM2	chr19:10001472-19634956	0.4078931
CRTC1	chr19:10001472-19634956	0.4078931
BRD4	chr19:10001472-19634956	0.4078931
DNAJB1	chr19:10001472-19634956	0.4078931
JAK3	chr19:10001472-19634956	0.4078931

(continued)

Gene	Segment	Absolute_Diff
LYL1	chr19:10001472-19634956	0.4078931
PRKACA	chr19:10001472-19634956	0.4078931
SMARCA4	chr19:10001472-19634956	0.4078931
TPM4	chr19:10001472-19634956	0.4078931
CALR	chr19:10001472-19634956	0.4078931
ELL	chr19:10001472-19634956	0.4078931
KEAP1	chr19:10001472-19634956	0.4078931

VI. Genes with Double Hit

A double hit strongly suggests a relevant tumor-suppressor gene [10]. In this section, the list of genes with somatic SNV/indel(s) as well as SCNA and/or LOH events are reported.

Gene.Symbol	Protein.Change.s.	SCNA	LOH	DDR	Irino	TMZ	KEGG	CGC
C7orf65	NA	AMP (1.41)	-	no	no	no	no	no
FPR1	p.YLVF30del	AMP (1.34)	-	no	no	no	no	no
LIN28A	p.R61H	DEL (0.57)	-	no	no	no	no	no
NLRP4	p.P223L	AMP (1.33)	-	no	no	no	no	no
OCSTAMP	p.R395W	AMP (1.45)	-	no	no	no	no	no
PIK3CG	p.R839C	AMP (1.41)	-	no	no	no	no	no
PLCG1	p.I459S	AMP (1.45)	-	no	no	no	Pathways in Cancer	yes
RYR1	p.V1551I	AMP (1.3)	-	no	no	no	no	no
SLC44A2	p.C158F	-	0.407893106705203	no	no	no	no	no
WFDC13	p.R78H	AMP (1.45)	-	no	no	no	no	no

VII. Tumor Heterogeneity Analysis

THetA (Tumor Heterogeneity Analysis) is an algorithm that estimates the tumor purity and clonal/subclonal copy number aberrations directly from high-throughput DNA sequencing data.

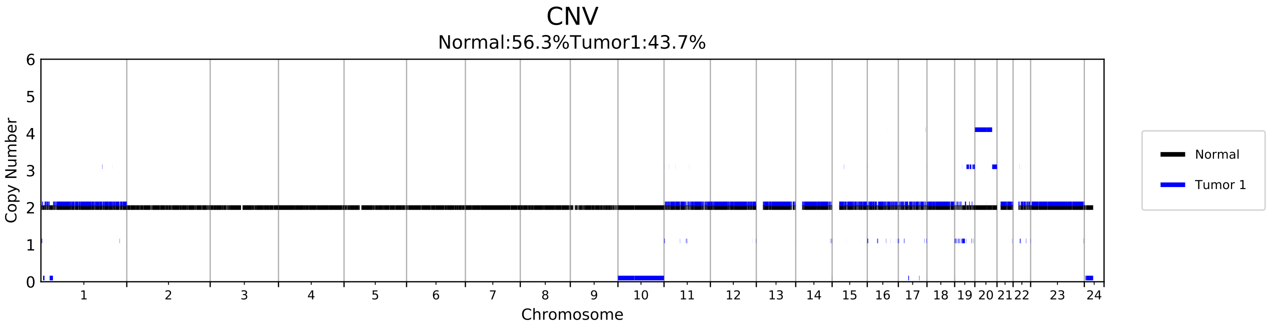

[10] Vogelstein B, Papadopoulos N, Velculescu VE, Zhou S, Diaz LA, Kinzler KW. Cancer genome landscapes. Science. 2013;339(6127):1546-58.

VIII. Mutational Signatures

Somatic mutations in cancer genomes are caused by multiple mutational processes each of which generates a characteristic mutational signature (i.e. a distinct pattern of substitution types in specific sequence contexts) [11]. Importantly, recent studies show that mutational signatures could have significant clinical impact in certain tumor types [12-13].

Using DeConstructSigs, the weights of Mutational Signatures v3 (May 2019) from COSMIC within this tumor's exome are estimated. This section presents the mutational signatures detected in this tumor.

[11] Ludmil B Alexandrov, Jaegil Kim, Nicholas J Haradhvala, Mi Ni Huang, Alvin WT Ng, Yang Wu, Arnoud Boot, Kyle R Covington, Dmitry A Gordenin, Erik N Bergstrom, S M Ashiqul Islam, Nuria Lopez-Bigas, Leszek J Klimczak, John R McPherson, Sandro Morganella, Radhakrishnan Sabarinathan, David A Wheeler, Ville Mustonen, the PCAWG Mutational Signatures Working Group, Gad Getz, Steven G Rozen, Michael R Stratton. The Repertoire of Mutational Signatures in Human Cancer. bioRxiv (2019). doi: 10.1101/322859

[12] Dong F, Davineni PK, Howitt BE, Beck AH. A BRCA1/2 Mutational Signature and Survival in Ovarian High-Grade Serous Carcinoma. Cancer Epidemiol Biomarkers Prev. 2016;25(11):1511-1516.

[13] Secrier M, Li X, De silva N, et al. Mutational signatures in esophageal adenocarcinoma define etiologically distinct subgroups with therapeutic relevance. Nat Genet. 2016;48(10):1131-41.

NOT−0086

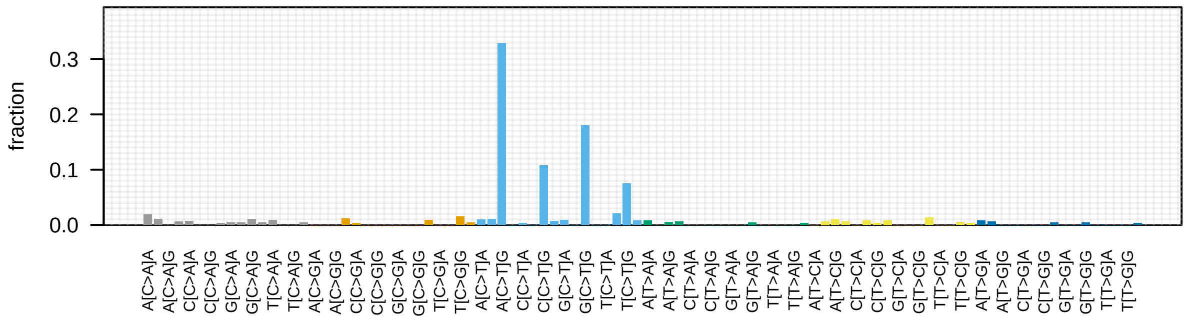

SBS1 : 0.735 & SBS50 : 0.138

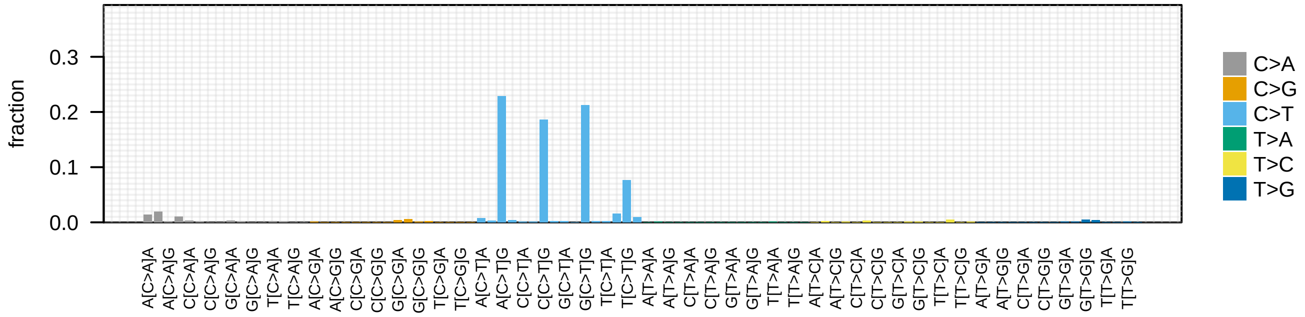

error = 0.138

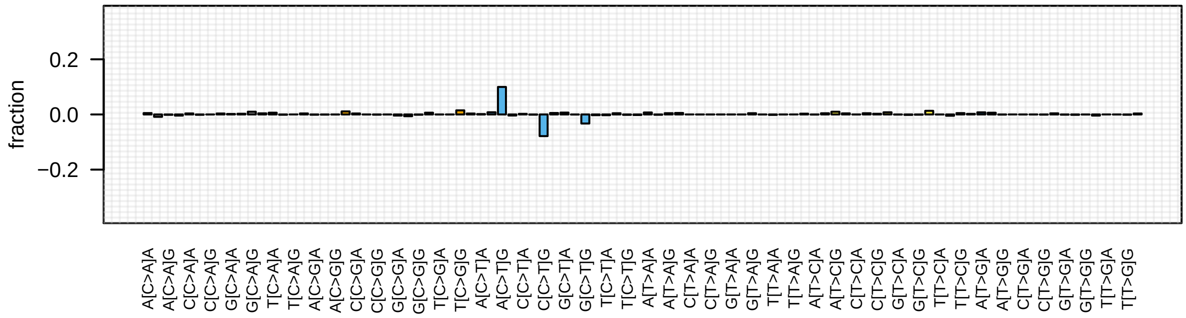

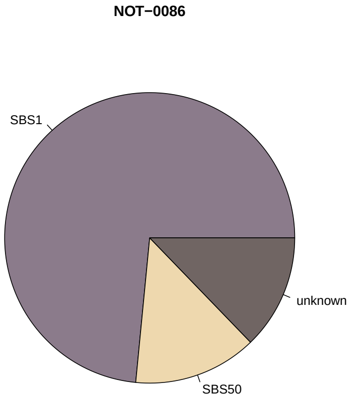

	freq	Proposed.aetiology	Associated.mutation.classes.and.signatures	Comments
SBS1	0.73	An endogenous mutational process initiated by spontaneous or enzymatic deamination of 5-methylcytosine to thymine which generates G:T mismatches in double stranded DNA. Failure to detect and remove these mismatches prior to DNA replication results in fixation of the T substitution for C.	The activity of SBS1 is closely correlated with the activity of SBS5 within many types of cancer. However, between cancer types, mutation burdens of SBS1 and SBS5 do not clearly correlate consistent with them being due to different underlying processes.	Signature SBS1 is clock-like in that the number of mutations in most cancers and normal cells correlates with the age of the individual. Rates of acquisition of Signature SBS1 mutations over time differ markedly between different cancer types and different normal cell types. These differences correlate with estimated rates of stem cell division in different tissues and Signature SBS1 may therefore be a cell division/mitotic clock.
SBS50	0.14	Possible sequencing artefact.	Possible sequencing artefact.	SBS50 was found in cancer samples that were subsequently blacklisted for poor quality of sequencing data.

IX. pathfindR - KEGG Pathway Enrichment Analysis

Pathway enrichment analysis is frequently utilized for studying the mechanisms underlying oncological processes. For this analysis KEGG pathway enrichment analysis was performed using the active-subnetwork-oriented enrichment approach of `pathfindR`. `pathfindR` leverages interaction information from a protein-protein interaction network (PIN) to identify distinct active subnetworks [14] and then perform enrichment analyses on these subnetworks.

IX. a. Enrichment Results for High-impact Somatic SNV/indels

Genes that harbor non-synonymous mutations with a VAF > 5% and not in FLAGS are included in the enrichment analysis.

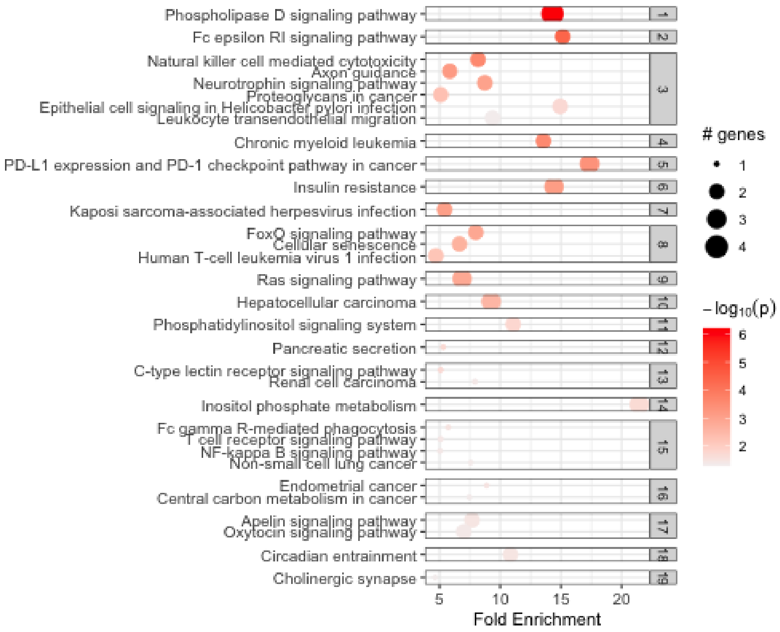

[14]An active subnetwork can be defined as a group of interconnected genes in a PIN that predominantly consists of significantly altered genes

ID	Term_Description	Fold_Enrichment	occurrence	support	lowest_p	Somatic SNV/indel	Up_regulated
hsa04072	Phospholipase D signaling pathway	14.303523	1	0.0821918	0.0000006	0.0000006	PTPN11, PLCG1, MS4A2, PIK3CG
hsa04664	Fc epsilon RI signaling pathway	15.144907	1	0.0410959	0.0000390	0.0000390	MS4A2, PLCG1
hsa04650	Natural killer cell mediated cytotoxicity	8.173442	1	0.0273973	0.0002530	0.0002530	PTPN11, PLCG1
hsa05220	Chronic myeloid leukemia	13.550706	1	0.0136986	0.0002722	0.0002722	PTPN11, TGFBR2
hsa05235	PD-L1 expression and PD-1 checkpoint pathway in cancer	17.357084	1	0.0821918	0.0004392	0.0004392	PTEN, PTPN11, PLCG1
hsa04360	Axon guidance	5.851441	1	0.0136986	0.0006929	0.0006929	PLCG1, PTPN11
hsa04931	Insulin resistance	14.437201	1	0.0684932	0.0007661	0.0007661	PTPN11, TBC1D4, PTEN
hsa05167	Kaposi sarcoma-associated herpesvirus infection	5.420282	1	0.0136986	0.0008724	0.0008724	PIK3CG, PLCG1
hsa04722	Neurotrophin signaling pathway	8.727573	1	0.0136986	0.0010290	0.0010290	PLCG1, PTPN11
hsa04068	FoxO signaling pathway	7.983362	1	0.0136986	0.0013458	0.0013458	TGFBR2, PTEN
hsa04014	Ras signaling pathway	6.865691	1	0.0410959	0.0014506	0.0014506	PTPN11, PLCG1, NF1
hsa04218	Cellular senescence	6.644217	1	0.0410959	0.0023373	0.0023373	TGFBR2, PTEN
hsa05225	Hepatocellular carcinoma	9.250183	1	0.0273973	0.0029236	0.0029236	PLCG1, PTEN, TGFBR2
hsa05205	Proteoglycans in cancer	5.098285	1	0.0273973	0.0051708	0.0051708	PTPN11, PLCG1
hsa05166	Human T-cell leukemia virus 1 infection	4.702528	1	0.0136986	0.0065850	0.0065850	PTEN, TGFBR2
hsa04070	Phosphatidylinositol signaling system	11.073695	1	0.0136986	0.0175722	0.0175722	PTEN, PLCG1
hsa04972	Pancreatic secretion	5.308524	1	0.0136986	0.0191224	0.0191224	CLCA1
hsa05120	Epithelial cell signaling in Helicobacter pylori infection	14.925415	1	0.0684932	0.0192466	0.0192466	PTPN11, PLCG1
hsa04625	C-type lectin receptor signaling pathway	5.098285	1	0.0136986	0.0207380	0.0207380	PTPN11
hsa00562	Inositol phosphate metabolism	21.455285	1	0.0410959	0.0209654	0.0209654	PIK3CG, PTEN, PLCG1
hsa04666	Fc gamma R-mediated phagocytosis	5.721409	1	0.0136986	0.0328133	0.0328133	PLCG1
hsa05213	Endometrial cancer	8.878049	1	0.0136986	0.0338067	0.0338067	PTEN
hsa04371	Apelin signaling pathway	7.685475	1	0.0136986	0.0365550	0.0365550	RYR1, PIK3CG
hsa04713	Circadian entrainment	10.840565	1	0.0136986	0.0365706	0.0365706	RYR1, GRIA4
hsa04660	T cell receptor signaling pathway	5.098285	1	0.0136986	0.0413463	0.0413463	PLCG1
hsa04064	NF-kappa B signaling pathway	5.048302	1	0.0136986	0.0421705	0.0421705	PLCG1
hsa05211	Renal cell carcinoma	7.921951	1	0.0273973	0.0425020	0.0425020	PTPN11
hsa04921	Oxytocin signaling pathway	7.005807	1	0.0136986	0.0440029	0.0440029	RYR1, PIK3CG
hsa05223	Non-small cell lung cancer	7.572453	1	0.0136986	0.0465302	0.0465302	PLCG1
hsa05230	Central carbon metabolism in cancer	7.462708	1	0.0136986	0.0479131	0.0479131	PTEN
hsa04670	Leukocyte transendothelial migration	9.362306	1	0.0273973	0.0490554	0.0490554	PTPN11, PLCG1
hsa04725	Cholinergic synapse	4.638980	1	0.0136986	0.0499523	0.0499523	PIK3CG

IX. b. Enrichment Results for High-impact SCNA

Genes that harbor homozygous deletion or multi-copy amplification (3+) are included in the enrichment analysis.

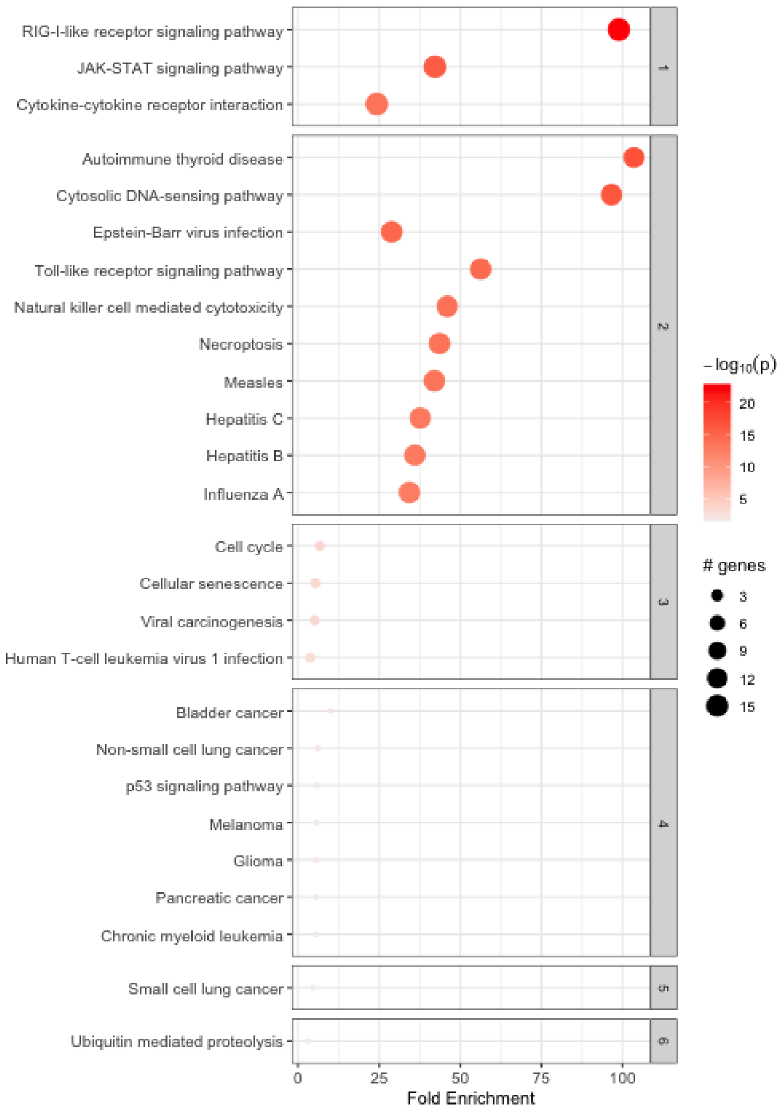

ID	Term_Description	Fold_Enrichment	occurrence	support	lowest_p	Amplification	Deletion	Down_regulated
hsa04622	RIG-I-like receptor signaling pathway	98.856307	1	0.2121212	0.0000000	0.0000000	NA	IFNA1, IFNA2, IFNA4, IFNA5, IFNA6, IFNA7, IFNA8, IFNA10, IFNA13, IFNA14, IFNA16, IFNA17, IFNA21, IFNB1, IFNW1, IFNE
hsa05320	Autoimmune thyroid disease	103.490196	1	0.1818182	0.0000000	0.0000000	NA	IFNA1, IFNA2, IFNA4, IFNA5, IFNA6, IFNA7, IFNA8, IFNA10, IFNA13, IFNA14, IFNA16, IFNA17, IFNA21
hsa04623	Cytosolic DNA-sensing pathway	96.590850	1	0.1818182	0.0000000	0.0000000	NA	IFNA1, IFNA2, IFNA4, IFNA5, IFNA6, IFNA7, IFNA8, IFNA10, IFNA13, IFNA14, IFNA16, IFNA17, IFNA21, IFNB1
hsa04630	JAK-STAT signaling pathway	42.187086	1	0.1515152	0.0000000	0.0000000	NA	IFNA1, IFNA2, IFNA4, IFNA5, IFNA6, IFNA7, IFNA8, IFNA10, IFNA13, IFNA14, IFNA16, IFNA17, IFNA21, IFNB1, IFNE, IFNW1
hsa05169	Epstein-Barr virus infection	28.833089	1	0.1515152	0.0000000	0.0000000	NA	IFNA1, IFNA2, IFNA4, IFNA5, IFNA6, IFNA7, IFNA8, IFNA10, IFNA13, IFNA14, IFNA16, IFNA17, IFNA21, IFNB1
hsa04620	Toll-like receptor signaling pathway	56.266514	1	0.1818182	0.0000000	0.0000000	NA	IFNA1, IFNA2, IFNA4, IFNA5, IFNA6, IFNA7, IFNA8, IFNA10, IFNA13, IFNA14, IFNA16, IFNA17, IFNA21, IFNB1
hsa04650	Natural killer cell mediated cytotoxicity	45.995643	1	0.1515152	0.0000000	0.0000000	NA	IFNA1, IFNA2, IFNA4, IFNA5, IFNA6, IFNA7, IFNA8, IFNA10, IFNA13, IFNA14, IFNA16, IFNA17, IFNA21, IFNB1
hsa04060	Cytokine-cytokine receptor interaction	24.261438	1	0.1212121	0.0000000	0.0000000	NA	IFNA1, IFNA2, IFNA4, IFNA5, IFNA6, IFNA7, IFNA8, IFNA10, IFNA13, IFNA14, IFNA16, IFNA17, IFNA21, IFNB1, IFNW1, IFNE
hsa04217	Necroptosis	43.574819	1	0.1515152	0.0000000	0.0000000	NA	IFNA1, IFNA2, IFNA4, IFNA5, IFNA6, IFNA7, IFNA8, IFNA10, IFNA13, IFNA14, IFNA16, IFNA17, IFNA21, IFNB1
hsa05162	Measles	41.996022	1	0.1515152	0.0000000	0.0000000	NA	IFNA1, IFNA2, IFNA4, IFNA5, IFNA6, IFNA7, IFNA8, IFNA10, IFNA13, IFNA14, IFNA16, IFNA17, IFNA21, IFNB1
hsa05160	Hepatitis C	37.632799	1	0.1515152	0.0000000	0.0000000	NA	IFNA1, IFNA2, IFNA4, IFNA5, IFNA6, IFNA7, IFNA8, IFNA10, IFNA13, IFNA14, IFNA16, IFNA17, IFNA21, IFNB1
hsa05161	Hepatitis B	35.996590	1	0.1515152	0.0000000	0.0000000	NA	IFNA1, IFNA2, IFNA4, IFNA5, IFNA6, IFNA7, IFNA8, IFNA10, IFNA13, IFNA14, IFNA16, IFNA17, IFNA21, IFNB1
hsa05164	Influenza A	34.292609	1	0.1515152	0.0000000	0.0000000	NA	IFNA1, IFNA2, IFNA4, IFNA5, IFNA6, IFNA7, IFNA8, IFNA10, IFNA13, IFNA14, IFNA16, IFNA17, IFNA21, IFNB1
hsa04621	NOD-like receptor signaling pathway	34.090888	1	0.1515152	0.0000000	0.0000000	NA	IFNA1, IFNA2, IFNA4, IFNA5, IFNA6, IFNA7, IFNA8, IFNA10, IFNA13, IFNA14, IFNA16, IFNA17, IFNA21, IFNB1
hsa05152	Tuberculosis	32.558713	1	0.1515152	0.0000000	0.0000000	NA	IFNA1, IFNA2, IFNA4, IFNA5, IFNA6, IFNA7, IFNA8, IFNA10, IFNA13, IFNA14, IFNA16, IFNA17, IFNA21, IFNB1
hsa05167	Kaposi sarcoma-associated herpesvirus infection	30.502374	1	0.1515152	0.0000000	0.0000000	NA	IFNA1, IFNA2, IFNA4, IFNA5, IFNA6, IFNA7, IFNA8, IFNA10, IFNA13, IFNA14, IFNA16, IFNA17, IFNA21, IFNB1
hsa05170	Human immunodeficiency virus 1 infection	28.409073	1	0.1515152	0.0000000	0.0000000	NA	IFNA1, IFNA2, IFNA4, IFNA5, IFNA6, IFNA7, IFNA8, IFNA10, IFNA13, IFNA14, IFNA16, IFNA17, IFNA21, IFNB1
hsa05163	Human cytomegalovirus infection	27.970323	1	0.1515152	0.0000000	0.0000000	NA	IFNA1, IFNA2, IFNA4, IFNA5, IFNA6, IFNA7, IFNA8, IFNA10, IFNA13, IFNA14, IFNA16, IFNA17, IFNA21, IFNB1, CDKN2A
hsa04110	Cell cycle	6.676787	1	0.0909091	0.0002410	0.0002410	NA	CDKN2A, CDKN2B
hsa04218	Cellular senescence	5.341430	1	0.0606061	0.0004725	0.0004725	NA	CDKN2B, CDKN2A
hsa05203	Viral carcinogenesis	5.048302	1	0.0303030	0.0005602	0.0005602	NA	CDKN2A, CDKN2B
hsa05166	Human T-cell leukemia virus 1 infection	3.780464	1	0.0303030	0.0013374	0.0013374	NA	CDKN2B, CDKN2A
hsa05219	Bladder cancer	10.096604	1	0.0303030	0.0067392	0.0067392	NA	CDKN2A
hsa05223	Non-small cell lung cancer	6.087659	1	0.0303030	0.0186899	0.0186899	NA	CDKN2A
hsa04115	p53 signaling pathway	5.749455	1	0.0303030	0.0209654	0.0209654	NA	CDKN2A
hsa05218	Melanoma	5.749455	1	0.0303030	0.0209654	0.0209654	NA	CDKN2A
hsa05214	Glioma	5.519477	1	0.0303030	0.0227574	0.0227574	NA	CDKN2A
hsa05212	Pancreatic cancer	5.446852	1	0.0303030	0.0233710	0.0233710	NA	CDKN2A
hsa05220	Chronic myeloid leukemia	5.446852	1	0.0303030	0.0233710	0.0233710	NA	CDKN2A
hsa05222	Small cell lung cancer	4.499574	1	0.0303030	0.0342919	0.0342919	NA	CDKN2B
hsa04120	Ubiquitin mediated proteolysis	3.021612	1	0.0303030	0.0382128	0.0382128	NA	KLHL9
