# Supplementary material for: Sequential filtering for clinically relevant variants as a method for clinical interpretation of whole exome sequencing findings in glioma
Source: BMC Med Genomics. 2021 Feb 23;14:54. doi: 10.1186/s12920-021-00904-3 (PMC7903763; doi:10.1186/s12920-021-00904-3)

# Sequential Filtering for Clinically Relevant Variants as a Method for Clinical Interpretation of Whole Exome Sequencing Findings in Glioma

Ege Ülgen[1], Özge Can[2], Kaya Bilguvar[3, 4], Cemaliye Akyerli Boylu[5], Şirin Kılıçturgay Yüksel[5], Ayça Erşen Danyeli[6], O. Uğur Sezerman[1], M. Cengiz Yakıcıer[7], M Necmettin Pamir[8], Koray Özduman[8]

Acibadem Mehmet Ali Aydinlar University, School of Medicine, Departments of Biostatistics and Medical Informatics[1], Medical Biology[5], Pathology[6], Neurosurgery[8], Istanbul, Turkey

Acibadem Mehmet Ali Aydinlar University, Faculty of Engineering, Department of Medical Engineering[2], Istanbul, Turkey

Yale University, School of Medicine, Department of Genetics[3], New Haven, CT, USA

Yale Center for Genome Analysis[4], West Haven, CT, USA

Acibadem Mehmet Ali Aydinlar University, School of Arts and Sciences, Department of Molecular Biology[7] Istanbul, Turkey

**Supplementary Table 1:** Clinical characteristics of all analyses. "Analysis ID" indicates the id of the analyzed tumor-normal pair. "Patient ID" indicates the id of the patient whose tumor was analyzed. "Sample Type" indicates whether the tumor sample was fresh tumor tissue, snap-frozen in liquid nitrogen ("LiN2") or formaldehyde-fixed paraffin-embedded tissue ("FFPE"). "Op. Status" indicates whether this is the initial tumor ("primary") or a recurrent tumor ("recurrent"). "Sex" indicates the sex of the patient ("F" for female, "M" for male). "Age at Diagnosis" indicates the age of the patient when the tumor was diagnosed. "Anatomical Compartment" indicates the "compartment" where the tumor is predominantly localized ("GM" for gliomatosis, "HE" for hemispheric, "LI" for limbic, "BS" for brainstem, "CC" for corpus callosum, "CR" for cerebellar and "TH" for thalamic). "Pathological Diagnosis" and "WHO Grade" indicate the pathological diagnosis and the grade of the tumor based on the 2016 WHO classification of tumors of the central nervous system criteria. "IDH1/2" indicates whether the tumor has a somatic mutation in the genes *IDH1* or *IDH2* ("WT" indicates wild-type, otherwise amino acid change is reported). "TERT" indicates whether the tumor harbors a somatic mutation in the promoter of the gene *TERT* ("C250" or "C228") or the promoter is wild-type ("WT"). "ATRX" indicates whether the tumor has any somatic mutation in *ATRX* ("MUT") or not ("WT"). "H3.3" indicates whether the tumor has a somatic mutation in *H3F3A* ("G34R" or "K27M") or not ("WT"). "Death" indicates the survival status (0 if alive, 1 if dead). "OS" indicates the overall survival time in months. "N/A" indicates "not applicable/available".

| Analysis ID | Patient ID | Sample Type | Op. Status | Sex | Age at Diagnosis | Anatomical Compartment | Pathological Diagnosis | WHO Grade | IDH1/2 | TERT | ATRX | H3.3 | Death | OS |
|---|---|---|---|---|---|---|---|---|---|---|---|---|---|---|
| **NOT-0044** | NOT-0044 | FFPE | primary | F | 33 | HE | Glioblastoma, IDH-mutant | IV | R132H | WT | MUT | WT | 0 | 134 |
| **NOT-0045** | NOT-0045 | FFPE | primary | F | 38 | LI | Diffuse astrocytoma, IDH-mutant | II | R132H | WT | MUT | WT | 1 | 54 |
| **NOT-0046_TA** | NOT-0046 | FFPE | primary | M | 31 | TH | Glioblastoma, IDH wild type | IV | WT | WT | MUT | WT | 1 | 96 |
| **NOT-0046_TB** | NOT-0046 | LiN2 | recurrent | M | 31 | TH | Glioblastoma, IDH wild type | IV | WT | WT | MUT | WT | N/A | N/A |
| **NOT-0047** | NOT-0047 | LiN2 | recurrent | M | 49 | HE | Glioblastoma, IDH wild type | IV | WT | WT | WT | WT | 1 | 38 |
| **NOT-0048** | NOT-0048 | LiN2 | primary | F | 45 | HE | Glioblastoma, IDH wild type | IV | WT | WT | WT | WT | 1 | 17 |
| **NOT-0049** | NOT-0049 | LiN2 | primary | M | 20 | HE | Diffuse astrocytoma, IDH-mutant | II | R132H | WT | WT | WT | 0 | 56 |
| **NOT-0050** | NOT-0050 | LiN2 | recurrent | F | 63 | HE | Anaplastic astrocytoma, IDH mutant | III | R132H | WT | MUT | WT | 1 | 138 |
| **NOT-0051** | NOT-0051 | FFPE | primary | M | 48 | HE | Glioblastoma, IDH wild type | IV | WT | C228 | WT | WT | 0 | 28 |
| **NOT-0052** | NOT-0052 | FFPE | primary | F | 65 | LI | Anaplastic astrocytoma, IDH wild type | III | WT | C228 | WT | WT | 1 | 17 |
| **NOT-0053** | NOT-0053 | FFPE | recurrent | M | 36 | HE | Glioblastoma, IDH-mutant | IV | R132H | WT | WT | WT | 1 | 76 |
| **NOT-0054** | NOT-0054 | LiN2 | recurrent | F | 40 | HE | Glioblastoma, IDH wild type | IV | WT | C250 | WT | WT | 1 | 34 |
| **NOT-0055** | NOT-0055 | LiN2 | primary | M | 23 | GM | Diffuse midline glioma, H3.3 K27M mutant | IV | WT | WT | MUT | K27M | 1 | 8 |
| **NOT-0056** | NOT-0056 | LiN2 | primary | F | 67 | LI | Glioblastoma, IDH wild type | IV | WT | C228 | WT | WT | 1 | 18 |

| | | | | | | | | | | | | | | |
|---|---|---|---|---|---|---|---|---|---|---|---|---|---|---|
| **NOT-0057** | NOT-0057 | LiN2 | primary | F | 62 | HE | Glioblastoma, IDH wild type | IV | WT | C228 | WT | WT | 1 | 19 |
| **NOT-0058** | NOT-0058 | FFPE | primary | F | 71 | HE | Glioblastoma, IDH wild type | IV | WT | C228 | WT | WT | 0 | 34 |
| **NOT-0059** | NOT-0059 | FFPE | primary | F | 76 | HE | Glioblastoma, IDH wild type | IV | WT | WT | WT | WT | 1 | 31 |
| **NOT-0060** | NOT-0060 | FFPE | primary | M | 48 | LI | Glioblastoma, IDH wild type | IV | WT | C250 | WT | WT | 1 | 17 |
| **NOT-0061** | NOT-0061 | FFPE | primary | M | 55 | HE | Glioblastoma, IDH wild type | IV | WT | C250 | WT | WT | 0 | 30 |
| **NOT-0062** | NOT-0062 | FFPE | primary | M | 66 | HE | Glioblastoma, IDH wild type | IV | WT | C250 | WT | WT | 0 | 32 |
| **NOT-0063** | NOT-0063 | LiN2 | primary | M | 37 | GM | Glioblastoma, IDH wild type | IV | WT | WT | MUT | G34R | 1 | 9 |
| **NOT-0064** | NOT-0064 | LiN2 | primary | F | 48 | HE | Glioblastoma, IDH wild type | IV | WT | C228 | WT | WT | 1 | 12 |
| **NOT-0065** | NOT-0065 | LiN2 | primary | M | 59 | HE | Glioblastoma, IDH wild type | IV | WT | C228 | WT | WT | 1 | 47 |
| **NOT-0066** | NOT-0066 | LiN2 | primary | F | 69 | HE | Glioblastoma, IDH wild type | IV | WT | C228 | WT | WT | 1 | 14 |
| **NOT-0067** | NOT-0067 | LiN2 | primary | F | 51 | HE | Glioblastoma, IDH wild type | IV | WT | WT | WT | WT | 0 | 27 |
| **NOT-0068** | NOT-0068 | LiN2 | primary | M | 64 | GM | Diffuse astrocytoma, IDH-wild type | II | WT | WT | MUT | WT | 1 | 17 |
| **NOT-0069_TA** | NOT-0069 | FFPE | primary | M | 46 | HE | Glioblastoma, IDH wild type | IV | WT | C228 | WT | WT | 1 | 29 |
| **NOT-0069_TB** | NOT-0069 | LiN2 | recurrent | M | 46 | HE | Glioblastoma, IDH wild type | IV | WT | C228 | WT | WT | N/A | N/A |
| **NOT-0070** | NOT-0070 | FFPE | primary | M | 46 | HE | Glioblastoma, IDH wild type | IV | WT | C250 | WT | WT | 1 | 25 |
| **NOT-0071** | NOT-0071 | LiN2 | primary | M | 47 | HE | Glioblastoma, IDH wild type | IV | WT | WT | WT | WT | 1 | 8 |
| **NOT-0072** | NOT-0072 | FFPE | recurrent | M | 25 | HE | Anaplastic astrocytoma, IDH mutant | III | R132H | WT | MUT | WT | 1 | 116 |
| **NOT-0073** | NOT-0073 | LiN2 | primary | M | 51 | HE | Glioblastoma, IDH wild type | IV | WT | C228 | WT | WT | 1 | 18 |
| **NOT-0074** | NOT-0074 | LiN2 | primary | M | 45 | BS | Diffuse midline glioma, H3.3 K27M mutant | IV | WT | WT | MUT | K27M | 0 | 14 |
| **NOT-0075_TA** | NOT-0075 | LiN2 | primary | F | 49 | CR | Anaplastic astrocytoma, IDH wild type | III | WT | WT | MUT | WT | 1 | 34 |
| **NOT-0075_TB** | NOT-0075 | LiN2 | recurrent | F | 49 | CR | Glioblastoma, IDH wild type | IV | WT | WT | MUT | WT | N/A | N/A |
| **NOT-0076** | NOT-0076 | LiN2 | primary | M | 51 | TH | Glioblastoma, IDH wild type | IV | WT | C228 | WT | WT | 1 | 9 |
| **NOT-0077** | NOT-0077 | FFPE | primary | M | 36 | HE | Anaplastic astrocytoma, IDH mutant | III | R132H | WT | MUT | WT | 0 | 143 |
| **NOT-0078** | NOT-0078 | LiN2 | primary | F | 52 | HE | Diffuse astrocytoma, WHO grade II, IDH wild type | II | WT | WT | MUT | WT | 0 | 17 |
| **NOT-0079** | NOT-0079 | LiN2 | primary | M | 40 | HE | Glioblastoma, IDH wild type | IV | WT | C228 | WT | WT | 0 | 15 |
| **NOT-0082** | NOT-0082 | FFPE | primary | M | 68 | GM | Anaplastic astrocytoma, WHO grade III, IDH-wild type | III | WT | C228 | WT | WT | 0 | 18 |
| **NOT-0083** | NOT-0083 | FFPE | primary | F | 41 | CC | Glioblastoma, IDH wild type | IV | WT | WT | MUT | WT | 0 | 20 |
| **NOT-0084** | NOT-0084 | LiN2 | primary | M | 54 | HE | Glioblastoma, IDH wild type | IV | WT | WT | WT | WT | 0 | 10 |
| **NOT-0085** | NOT-0085 | LiN2 | primary | M | 59 | HE | Glioblastoma, IDH wild type | IV | WT | C250 | WT | WT | 0 | 5 |

| | | | | | | | | | | | | | | |
|---|---|---|---|---|---|---|---|---|---|---|---|---|---|---|
| **NOT-0086** | NOT-0086 | FFPE | primary | M | 53 | HE | Glioblastoma, IDH wild type | IV | WT | C250 | WT | WT | 0 | 6 |
| **NOT-0087** | NOT-0087 | FFPE | primary | M | 63 | HE | Glioblastoma, IDH wild type | IV | WT | C250 | WT | WT | 0 | 8 |
| **NOT-0088_TA** | NOT-0088 | LiN2 | primary | F | 34 | CR | Anaplastic astrocytoma with piloid features, IDH wild type | III | WT | WT | WT | WT | 1 | 21 |
| **NOT-0088_TB** | NOT-0088 | LiN2 | recurrent | F | 34 | CR | Glioblastoma, IDH wild type | IV | WT | WT | WT | WT | N/A | N/A |
| **NOT-0089** | NOT-0089 | LiN2 | primary | M | 62 | HE | Glioblastoma, IDH wild type | IV | WT | C228 | WT | WT | 0 | 3 |
| **NOT-0090_TA** | NOT-0090 | LiN2 | primary | M | 28 | HE | Glioblastoma, IDH wild type | IV | WT | WT | MUT | WT | 0 | 119 |
| **NOT-0090_TB** | NOT-0090 | LiN2 | recurrent | M | 28 | HE | Glioblastoma, IDH wild type | IV | WT | WT | MUT | WT | N/A | N/A |
| **NOT-0091** | NOT-0091 | FFPE | primary | F | 48 | HE | Glioblastoma, IDH wild type | IV | WT | C228 | WT | WT | 0 | 2 |
| **NOT-0092_TA** | NOT-0092 | LiN2 | primary | M | 51 | HE | Glioblastoma, IDH wild type | IV | WT | WT | WT | WT | 0 | 18 |
| **NOT-0092_TB** | NOT-0092 | LiN2 | recurrent | M | 51 | HE | Glioblastoma, IDH wild type | IV | WT | WT | WT | WT | N/A | N/A |
| **NOT-0093** | NOT-0093 | LiN2 | primary | F | 38 | HE | Diffuse midline glioma, H3.3 K27M mutant | IV | WT | WT | WT | K27M | 0 | 2 |
| **NOT-0094** | NOT-0094 | FFPE | primary | M | 64 | HE | Glioblastoma, IDH wild type | IV | WT | C250 | WT | WT | 0 | 2 |
| **NOT-0097** | NOT-0097 | FFPE | primary | F | 60 | HE | Glioblastoma, IDH wild type | 4 | WT | C228 | WT | WT | 0 | 5 |
| **NOT-0098** | NOT-0098 | LiN2 | primary | F | 38 | TH | Diffuse midline glioma, H3.3 K27M mutant | 4 | WT | C228 | WT | K27M | 0 | 2 |
| **NOT-0099** | NOT-0099 | WT | primary | M | 49 | HE | Glioblastoma, IDH wild type | 4 | WT | C228 | WT | WT | 0 | 1 |

**Supplementary Table 2:** Detailed sequencing quality information for all samples. "Sample Name" indicates the name of the sample (in the format "Patient ID_tumor" for tumor samples and "Patient ID_normal" for blood samples). "≥1X", "≥5X", "≥10X", "≥25X", "≥50X" and "≥100X" indicate the percentage of bases that has a coverage greater than or equal to 1, 5, 10, 25, 50 and 100, respectively.

| Sample Name | Type | Read Length | Read Type | Mean Coverage | ≥1X | ≥5X | ≥10X | ≥25X | ≥50X | ≥100X | Estimated Cross Sample Contamination | Estimated Normal in Tumor Contamination |
|---|---|---|---|---|---|---|---|---|---|---|---|---|
| **NOT-0044-blood** | Normal | 74 | Paired-End | 109.96 | 99.5% | 98.2% | 96.5% | 89.9% | 75.1% | 45.8% | N/A | N/A |
| **NOT-0044-tumor** | Tumor | 74 | Paired-End | 235.05 | 99.1% | 98.0% | 96.9% | 93.7% | 87.6% | 73.4% | 0.14% | 61.65% |
| **NOT-0045-blood** | Normal | 74 | Paired-End | 168.35 | 98.7% | 97.1% | 95.5% | 90.5% | 81.2% | 62.4% | N/A | N/A |
| **NOT-0045-tumor** | Tumor | 74 | Paired-End | 274.45 | 99.2% | 98.2% | 97.4% | 95.0% | 90.6% | 79.7% | 0.13% | 67.73% |
| **NOT-0046-blood** | Normal | 76 | Paired-End | 90.14 | 99.9% | 99.8% | 99.5% | 97.3% | 74.4% | 28.3% | N/A | N/A |
| **NOT-0046-tumorA** | Tumor | 76 | Paired-End | 64.65 | 100.0% | 99.8% | 99.4% | 91.3% | 58.9% | 12.5% | 4.99% | 65.49% |
| **NOT-0046-tumorB** | Tumor | 76 | Paired-End | 198.38 | 100.0% | 99.9% | 99.8% | 99.4% | 97.0% | 77.7% | 0.01% | 62.69% |
| **NOT-0047-blood** | Normal | 76 | Paired-End | 106.72 | 99.9% | 99.2% | 98.1% | 92.7% | 77.1% | 44.0% | N/A | N/A |
| **NOT-0047-tumor** | Tumor | 76 | Paired-End | 194.5 | 99.9% | 99.6% | 99.0% | 96.7% | 90.2% | 71.2% | 0.25% | 38.03% |
| **NOT-0048-blood** | Normal | 76 | Paired-End | 96.05 | 99.7% | 98.9% | 97.6% | 90.9% | 72.9% | 38.3% | N/A | N/A |
| **NOT-0048-tumor** | Tumor | 76 | Paired-End | 201.38 | 99.8% | 99.4% | 98.9% | 96.9% | 91.1% | 73.1% | 0.12% | 36.52% |
| **NOT-0049-blood** | Normal | 76 | Paired-End | 96.82 | 99.9% | 99.1% | 97.8% | 91.2% | 73.1% | 38.5% | N/A | N/A |
| **NOT-0049-tumor** | Tumor | 76 | Paired-End | 172.11 | 99.9% | 99.5% | 98.8% | 96.1% | 88.2% | 66.7% | 0.75% | 58.36% |
| **NOT-0050-blood** | Normal | 76 | Paired-End | 78.92 | 99.7% | 99.5% | 99.1% | 95.6% | 67.5% | 20.1% | N/A | N/A |
| **NOT-0050-tumor** | Tumor | 76 | Paired-End | 99.34 | 99.7% | 99.5% | 99.3% | 96.9% | 79.3% | 33.5% | 0.16% | 62.68% |
| **NOT-0051-blood** | Normal | 76 | Paired-End | 76.94 | 99.9% | 99.7% | 99.2% | 94.7% | 65.7% | 19.0% | N/A | N/A |
| **NOT-0051-tumor** | Tumor | 76 | Paired-End | 136.83 | 99.9% | 99.9% | 99.7% | 98.9% | 93.9% | 57.5% | 0.10% | 87.69% |
| **NOT-0052-blood** | Normal | 76 | Paired-End | 51.29 | 99.6% | 98.6% | 95.8% | 76.2% | 37.2% | 7.2% | N/A | N/A |
| **NOT-0052-tumor** | Tumor | 76 | Paired-End | 106.18 | 99.7% | 99.5% | 99.2% | 97.4% | 82.0% | 36.8% | 0.29% | 66.89% |

| | | | | | | | | | | | | |
|---|---|---|---|---|---|---|---|---|---|---|---|---|
| **NOT-0053-blood** | Normal | 76 | Paired-End | 71.64 | 99.9% | 99.5% | 98.5% | 89.7% | 59.5% | 17.2% | N/A | N/A |
| **NOT-0053-tumor** | Tumor | 76 | Paired-End | 110.72 | 99.9% | 99.8% | 99.6% | 98.0% | 84.8% | 41.4% | 0.06% | 61.97% |
| **NOT-0054-blood** | Normal | 76 | Paired-End | 75.49 | 99.7% | 99.4% | 99.0% | 93.9% | 62.6% | 18.6% | N/A | N/A |
| **NOT-0054-tumor** | Tumor | 76 | Paired-End | 167.68 | 99.7% | 99.6% | 99.5% | 98.8% | 95.3% | 69.1% | 0.15% | 61.46% |
| **NOT-0055-blood** | Normal | 76 | Paired-End | 95.96 | 100.0% | 99.8% | 99.5% | 97.8% | 81.2% | 31.4% | N/A | N/A |
| **NOT-0055-tumor** | Tumor | 76 | Paired-End | 114.28 | 100.0% | 99.9% | 99.7% | 98.2% | 85.8% | 43.4% | 0.59% | 22.38% |
| **NOT-0056-blood** | Normal | 76 | Paired-End | 108.13 | 99.7% | 99.6% | 99.4% | 98.1% | 86.9% | 40.8% | N/A | N/A |
| **NOT-0056-tumor** | Tumor | 76 | Paired-End | 113.49 | 99.7% | 99.6% | 99.3% | 97.8% | 86.4% | 43.8% | 0.03% | 64.62% |
| **NOT-0057-blood** | Normal | 101 | Paired-End | 178.5 | 99.8% | 99.7% | 99.7% | 99.4% | 98.1% | 85.3% | N/A | N/A |
| **NOT-0057-tumor** | Tumor | 101 | Paired-End | 151.96 | 99.9% | 99.8% | 99.7% | 99.2% | 96.5% | 73.3% | 2.20% | 44.97% |
| **NOT-0058-blood** | Normal | 101 | Paired-End | 43.09 | 99.7% | 99.3% | 97.9% | 80.6% | 30.1% | 2.1% | N/A | N/A |
| **NOT-0058-tumor** | Tumor | 101 | Paired-End | 76.84 | 99.7% | 99.6% | 99.2% | 95.3% | 73.1% | 21.6% | 0.07% | 15.38% |
| **NOT-0059-blood** | Normal | 101 | Paired-End | 106.52 | 99.8% | 99.7% | 99.5% | 98.4% | 90.5% | 47.8% | N/A | N/A |
| **NOT-0059-tumor** | Tumor | 101 | Paired-End | 214.41 | 99.8% | 99.7% | 99.7% | 99.5% | 98.5% | 90.1% | 0.06% | 90.71% |
| **NOT-0060-blood** | Normal | 101 | Paired-End | 283.19 | 99.9% | 99.9% | 99.9% | 99.7% | 99.2% | 95.7% | N/A | N/A |
| **NOT-0060-tumor** | Tumor | 101 | Paired-End | 164.83 | 99.9% | 99.9% | 99.8% | 99.4% | 97.5% | 81.3% | 0.02% | 71.46% |
| **NOT-0061-blood** | Normal | 101 | Paired-End | 76.13 | 99.7% | 99.3% | 98.6% | 94.5% | 74.3% | 21.4% | N/A | N/A |
| **NOT-0061-tumor** | Tumor | 101 | Paired-End | 135.8 | 99.9% | 99.7% | 99.5% | 98.5% | 94.7% | 70.2% | 0.05% | 84.98% |
| **NOT-0062-blood** | Normal | 101 | Paired-End | 83.83 | 99.7% | 99.2% | 98.6% | 95.5% | 80.2% | 29.0% | N/A | N/A |
| **NOT-0062-tumor** | Tumor | 101 | Paired-End | 195.9 | 99.9% | 99.8% | 99.6% | 99.0% | 96.7% | 83.3% | 0.05% | 57.29% |
| **NOT-0063-blood** | Normal | 101 | Paired-End | 107.47 | 99.9% | 99.8% | 99.6% | 98.1% | 89.6% | 50.1% | N/A | N/A |
| **NOT-0063-tumor** | Tumor | 101 | Paired-End | 180.87 | 99.9% | 99.8% | 99.8% | 99.3% | 97.2% | 83.5% | 0.02% | 90.14% |
| **NOT-0064-blood** | Normal | 101 | Paired-End | 116.5 | 99.7% | 99.6% | 99.4% | 97.7% | 89.0% | 54.6% | N/A | N/A |
| **NOT-0064-tumor** | Tumor | 101 | Paired-End | 241.75 | 99.7% | 99.7% | 99.6% | 99.2% | 97.9% | 90.2% | 0.06% | 68.66% |
| **NOT-0065-blood** | Normal | 101 | Paired-End | 214.67 | 99.9% | 99.8% | 99.7% | 99.2% | 97.5% | 88.2% | N/A | N/A |
| **NOT-0065-tumor** | Tumor | 101 | Paired-End | 153.96 | 99.8% | 99.6% | 99.4% | 98.3% | 94.2% | 74.4% | 0.14% | 68.74% |
| **NOT-0066-blood** | Normal | 101 | Paired-End | 184.69 | 99.6% | 99.3% | 99.0% | 98.0% | 95.5% | 83.7% | N/A | N/A |

| | | | | | | | | | | | | |
|---|---|---|---|---|---|---|---|---|---|---|---|---|
| **NOT-0066-tumor** | Tumor | 101 | Paired-End | 258.52 | 99.7% | 99.6% | 99.5% | 99.0% | 97.5% | 90.4% | 0.06% | 19.03% |
| **NOT-0067-blood** | Normal | 101 | Paired-End | 145.18 | 99.7% | 99.6% | 99.5% | 98.9% | 96.4% | 76.7% | N/A | N/A |
| **NOT-0067-tumor** | Tumor | 101 | Paired-End | 216.85 | 99.7% | 99.7% | 99.6% | 99.2% | 97.8% | 88.6% | 0.05% | 51.40% |
| **NOT-0068-blood** | Normal | 101 | Paired-End | 111.95 | 99.9% | 99.8% | 99.6% | 98.2% | 90.2% | 51.9% | N/A | N/A |
| **NOT-0068-tumor** | Tumor | 101 | Paired-End | 241.62 | 99.9% | 99.9% | 99.8% | 99.5% | 98.3% | 91.1% | 0.07% | 72.08% |
| **NOT-0069-blood** | Normal | 101 | Paired-End | 126.46 | 99.9% | 99.8% | 99.7% | 98.8% | 94.2% | 64.3% | N/A | N/A |
| **NOT-0069-tumorA** | Tumor | 101 | Paired-End | 226.45 | 99.9% | 99.9% | 99.8% | 99.4% | 97.8% | 88.2% | 0.15% | 49.98% |
| **NOT-0069-tumorB** | Tumor | 101 | Paired-End | 235.95 | 99.9% | 99.9% | 99.8% | 99.6% | 98.6% | 92.6% | 0.17% | 75.01% |
| **NOT-0070-blood** | Normal | 101 | Paired-End | 137.9 | 99.9% | 99.8% | 99.6% | 98.7% | 94.6% | 69.6% | N/A | N/A |
| **NOT-0070-tumor** | Tumor | 101 | Paired-End | 129.13 | 99.9% | 99.8% | 99.6% | 98.2% | 88.2% | 55.1% | 0.14% | 47.03% |
| **NOT-0071-blood** | Normal | 101 | Paired-End | 101.49 | 99.9% | 99.8% | 99.7% | 98.4% | 89.2% | 44.3% | N/A | N/A |
| **NOT-0071-tumor** | Tumor | 101 | Paired-End | 171.36 | 99.9% | 99.9% | 99.8% | 99.4% | 97.3% | 79.8% | 0.11% | 86.47% |
| **NOT-0072-blood** | Normal | 101 | Paired-End | 120.83 | 99.9% | 99.9% | 99.8% | 99.1% | 93.8% | 59.3% | N/A | N/A |
| **NOT-0072-tumor** | Tumor | 101 | Paired-End | 194.13 | 99.9% | 99.9% | 99.9% | 99.7% | 98.7% | 89.6% | 0.63% | 67.87% |
| **NOT-0073-blood** | Normal | 101 | Paired-End | 112.45 | 99.9% | 99.8% | 99.6% | 98.5% | 93.2% | 57.4% | N/A | N/A |
| **NOT-0073-tumor** | Tumor | 101 | Paired-End | 191.59 | 99.9% | 99.8% | 99.7% | 99.3% | 97.9% | 87.6% | 0.05% | 74.85% |
| **NOT-0074-blood** | Normal | 101 | Paired-End | 165.7 | 99.9% | 99.9% | 99.8% | 99.3% | 97.6% | 83.6% | N/A | N/A |
| **NOT-0074-tumor** | Tumor | 101 | Paired-End | 143.94 | 99.9% | 99.8% | 99.7% | 99.2% | 96.7% | 76.5% | 0.06% | 71.79% |
| **NOT-0075-blood** | Normal | 101 | Paired-End | 126.33 | 99.7% | 99.7% | 99.6% | 98.9% | 95.9% | 67.4% | N/A | N/A |
| **NOT-0075-tumorA** | Tumor | 101 | Paired-End | 177.63 | 99.7% | 99.7% | 99.6% | 99.0% | 97.1% | 82.6% | 0.02% | 63.12% |
| **NOT-0075-tumorB** | Tumor | 101 | Paired-End | 300.12 | 99.8% | 99.7% | 99.7% | 99.5% | 98.6% | 93.2% | 0.06% | 60.76% |
| **NOT-0076-blood** | Normal | 101 | Paired-End | 104.59 | 99.9% | 99.8% | 99.7% | 98.6% | 90.4% | 46.9% | N/A | N/A |
| **NOT-0076-tumor** | Tumor | 101 | Paired-End | 182.62 | 99.9% | 99.9% | 99.8% | 99.4% | 97.4% | 82.8% | 0.05% | 57.18% |
| **NOT-0077-blood** | Normal | 101 | Paired-End | 108.39 | 99.9% | 99.8% | 99.7% | 98.7% | 91.3% | 50.2% | N/A | N/A |
| **NOT-0077-tumor** | Tumor | 101 | Paired-End | 144.97 | 99.9% | 99.8% | 99.6% | 98.0% | 90.4% | 64.7% | 0.23% | 51.82% |
| **NOT-0078-blood** | Normal | 101 | Paired-End | 91.05 | 99.7% | 99.6% | 99.5% | 98.1% | 86.5% | 33.2% | N/A | N/A |
| **NOT-0078-tumor** | Tumor | 101 | Paired-End | 171.51 | 99.8% | 99.7% | 99.6% | 99.4% | 98.3% | 86.0% | 0.04% | 65.63% |

| | | | | | | | | | | | | |
|---|---|---|---|---|---|---|---|---|---|---|---|---|
| **NOT-0079-blood** | Normal | 101 | Paired-End | 102.64 | 99.9% | 99.8% | 99.7% | 98.4% | 89.3% | 45.5% | N/A | N/A |
| **NOT-0079-tumor** | Tumor | 101 | Paired-End | 248.45 | 99.9% | 99.9% | 99.8% | 99.6% | 98.6% | 91.6% | 0.00% | 60.05% |
| **NOT-0082-blood** | Normal | 101 | Paired-End | 115.83 | 99.9% | 99.9% | 99.7% | 98.8% | 92.4% | 55.7% | N/A | N/A |
| **NOT-0082-tumor** | Tumor | 101 | Paired-End | 181.85 | 100.0% | 99.9% | 99.9% | 99.7% | 98.0% | 68.0% | 20.97% | 57.05% |
| **NOT-0083-blood** | Normal | 101 | Paired-End | 108.64 | 99.8% | 99.7% | 99.6% | 98.7% | 91.9% | 50.9% | N/A | N/A |
| **NOT-0083-tumor** | Tumor | 101 | Paired-End | 221.61 | 99.8% | 99.7% | 99.7% | 99.5% | 98.2% | 87.6% | 0.22% | 75.86% |
| **NOT-0084-blood** | Normal | 101 | Paired-End | 141.85 | 99.9% | 99.9% | 99.8% | 99.3% | 96.5% | 73.5% | N/A | N/A |
| **NOT-0084-tumor** | Tumor | 101 | Paired-End | 291.09 | 99.9% | 99.9% | 99.8% | 99.3% | 97.0% | 87.3% | 0.17% | 57.13% |
| **NOT-0085-blood** | Normal | 101 | Paired-End | 175.39 | 99.9% | 99.9% | 99.9% | 99.6% | 98.0% | 84.5% | N/A | N/A |
| **NOT-0085-tumor** | Tumor | 101 | Paired-End | 324.96 | 99.9% | 99.9% | 99.9% | 99.8% | 99.5% | 97.3% | 0.00% | 66.09% |
| **NOT-0086-blood** | Normal | 101 | Paired-End | 183.74 | 99.9% | 99.9% | 99.8% | 99.6% | 98.3% | 87.1% | N/A | N/A |
| **NOT-0086-tumor** | Tumor | 101 | Paired-End | 273.95 | 99.9% | 99.9% | 99.8% | 99.7% | 99.1% | 95.4% | 0.04% | 56.32% |
| **NOT-0087-blood** | Normal | 101 | Paired-End | 199.35 | 99.9% | 99.9% | 99.8% | 99.6% | 98.3% | 88.1% | N/A | N/A |
| **NOT-0087-tumor** | Tumor | 101 | Paired-End | 244.47 | 99.9% | 99.9% | 99.8% | 99.6% | 98.8% | 92.6% | 0.01% | 57.80% |
| **NOT-0088-blood** | Normal | 101 | Paired-End | 108.98 | 99.7% | 99.7% | 99.6% | 98.7% | 92.5% | 51.0% | N/A | N/A |
| **NOT-0088-tumorA** | Tumor | 101 | Paired-End | 230.47 | 99.8% | 99.7% | 99.7% | 99.4% | 98.6% | 93.1% | 0.08% | 65.17% |
| **NOT-0088-tumorB** | Tumor | 101 | Paired-End | 206.86 | 99.8% | 99.7% | 99.7% | 99.4% | 98.3% | 89.9% | 0.00% | 56.32% |
| **NOT-0089-blood** | Normal | 101 | Paired-End | 113.47 | 99.9% | 99.8% | 99.7% | 98.6% | 92.0% | 53.4% | N/A | N/A |
| **NOT-0089-tumor** | Tumor | 101 | Paired-End | 228.39 | 99.9% | 99.8% | 99.8% | 99.5% | 98.6% | 91.5% | 0.02% | 64.41% |
| **NOT-0090-blood** | Normal | 101 | Paired-End | 157.5 | 99.9% | 99.9% | 99.8% | 99.3% | 95.8% | 73.9% | N/A | N/A |
| **NOT-0090-tumorA** | Tumor | 101 | Paired-End | 223.73 | 99.9% | 99.9% | 99.9% | 99.7% | 98.7% | 91.8% | 0.01% | 53.15% |
| **NOT-0090-tumorB** | Tumor | 101 | Paired-End | 296.88 | 99.9% | 99.9% | 99.9% | 99.8% | 99.3% | 95.6% | 0.09% | 68.70% |
| **NOT-0091-blood** | Normal | 101 | Paired-End | 129.22 | 99.8% | 99.7% | 99.6% | 99.2% | 95.7% | 65.6% | N/A | N/A |
| **NOT-0091-tumor** | Tumor | 101 | Paired-End | 285.89 | 99.8% | 99.7% | 99.7% | 99.5% | 99.1% | 96.7% | 0.33% | 61.76% |
| **NOT-0092-blood** | Normal | 101 | Paired-End | 125.25 | 99.9% | 99.9% | 99.8% | 99.3% | 95.4% | 64.9% | N/A | N/A |
| **NOT-0092-tumorA** | Tumor | 101 | Paired-End | 280.11 | 99.9% | 99.9% | 99.8% | 99.6% | 98.8% | 93.8% | 0.02% | 46.76% |
| **NOT-0092-tumorB** | Tumor | 101 | Paired-End | 263.58 | 99.9% | 99.9% | 99.9% | 99.6% | 98.9% | 94.5% | 0.28% | 50.18% |

| | | | | | | | | | | | | |
|---|---|---|---|---|---|---|---|---|---|---|---|---|
| **NOT-0093-blood** | Normal | 101 | Paired-End | 146.6 | 99.7% | 99.7% | 99.6% | 99.1% | 96.6% | 73.8% | N/A | N/A |
| **NOT-0093-tumor** | Tumor | 101 | Paired-End | 223.67 | 99.8% | 99.7% | 99.7% | 99.5% | 98.9% | 92.8% | 0.05% | 90.07% |
| **NOT-0094-blood** | Normal | 101 | Paired-End | 108.4 | 99.9% | 99.9% | 99.8% | 98.9% | 93.0% | 51.6% | N/A | N/A |
| **NOT-0094-tumor** | Tumor | 101 | Paired-End | 150.73 | 99.9% | 99.9% | 99.7% | 99.0% | 95.5% | 73.2% | 0.03% | 61.31% |
| **NOT-0097-blood** | Normal | 101 | Paired-End | 119.54 | 99.7% | 99.7% | 99.6% | 98.6% | 91.9% | 56.4% | N/A | N/A |
| **NOT-0097-tumor** | Tumor | 101 | Paired-End | 217.22 | 99.7% | 99.6% | 99.5% | 99.0% | 97.8% | 89.2% | 0.02% | 48.98% |
| **NOT-0098-blood** | Normal | 101 | Paired-End | 75.15 | 99.7% | 99.6% | 99.2% | 95.9% | 73.8% | 18.5% | N/A | N/A |
| **NOT-0098-tumor** | Tumor | 101 | Paired-End | 214.25 | 99.8% | 99.7% | 99.7% | 99.4% | 98.3% | 89.3% | 0.00% | 52.45% |
| **NOT-0099-blood** | Normal | 101 | Paired-End | 137.87 | 99.9% | 99.8% | 99.8% | 99.1% | 95.1% | 67.8% | N/A | N/A |
| **NOT-0099-tumor** | Tumor | 101 | Paired-End | 257.72 | 99.9% | 99.9% | 99.9% | 99.6% | 98.5% | 92.0% | 0.02% | 84.96% |

**Figure S1: Percentages of clinical significances of variants reported in each germline variant category in all patients**

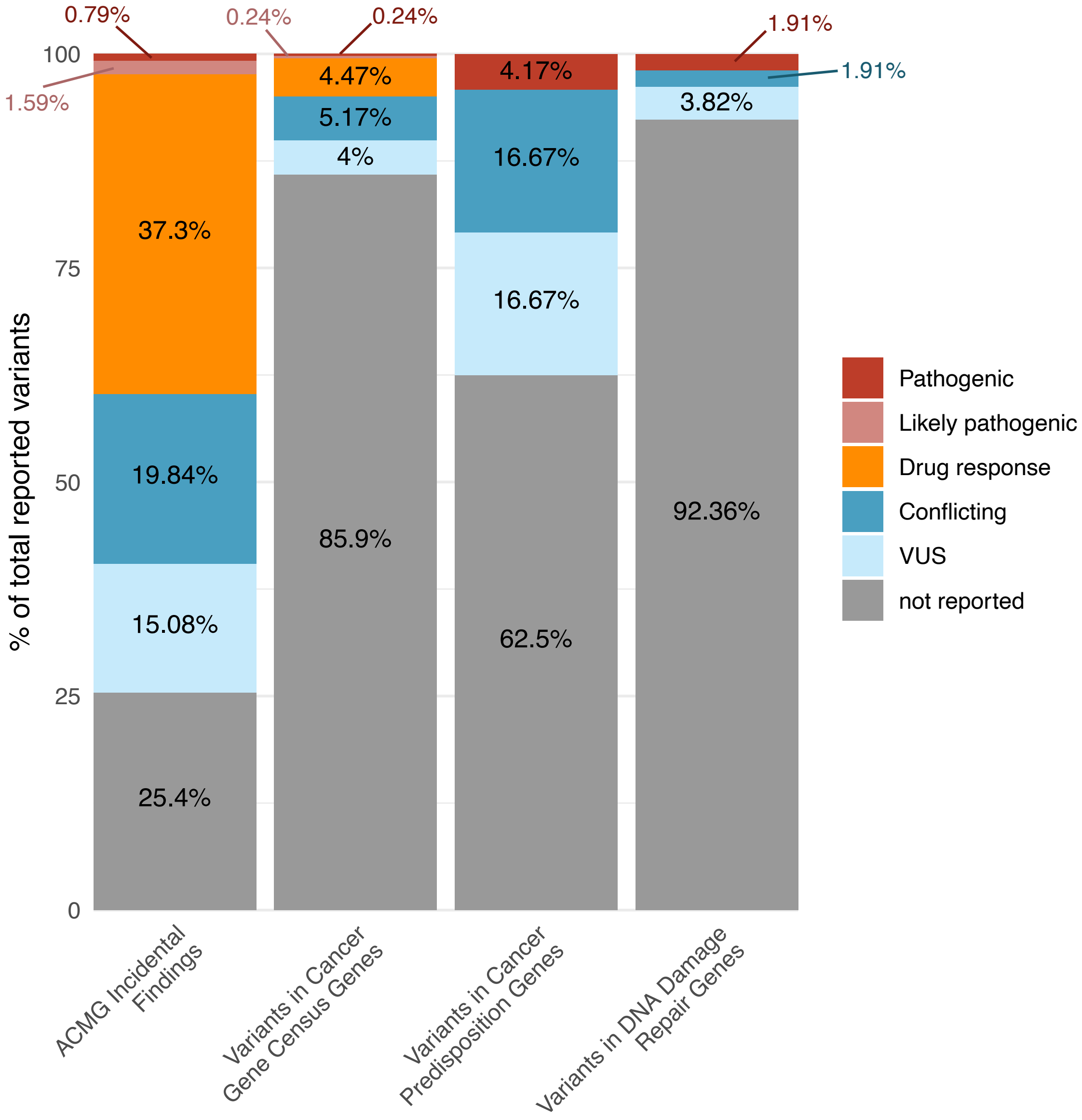

**Figure S2: Tile plot of chromosomal arm-level somatic copy-number alteration events in each tumor**

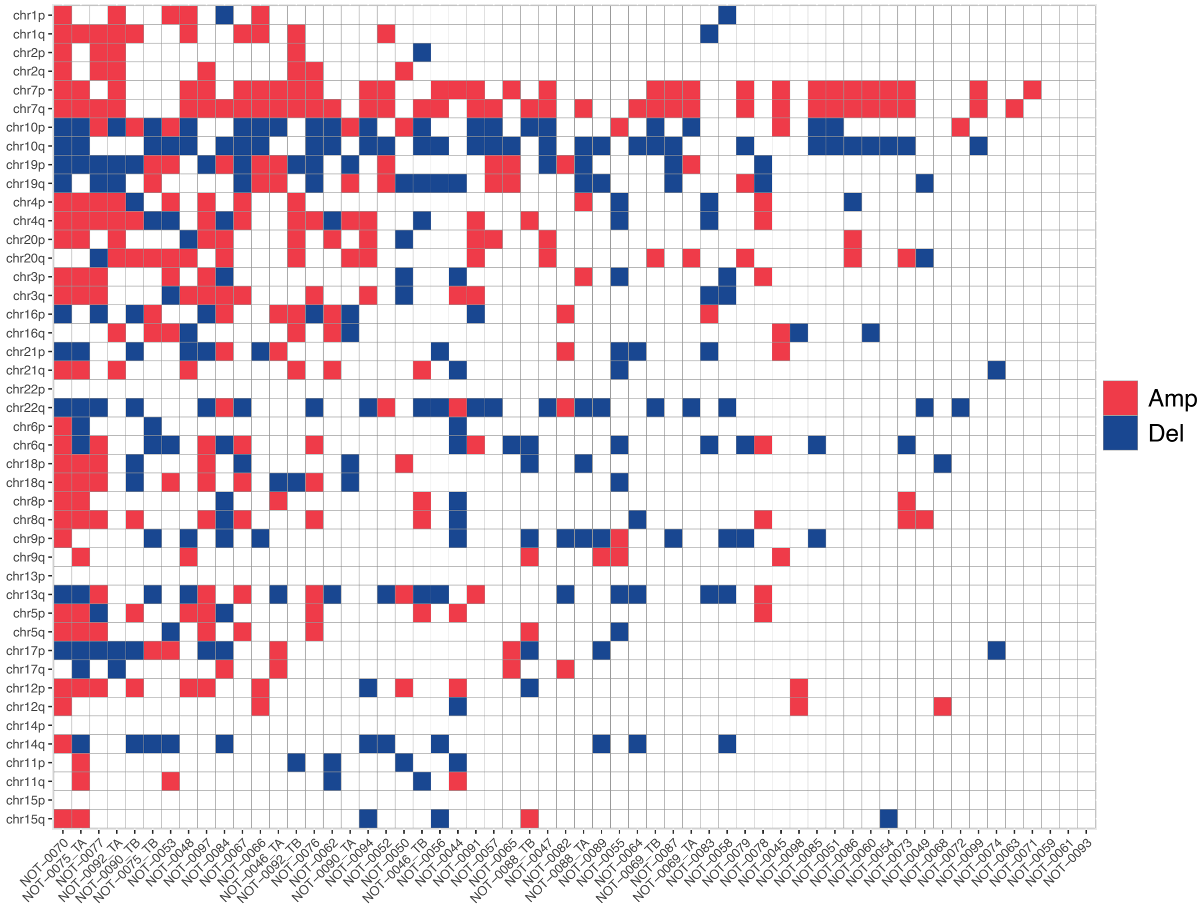

**Figure S3: Tumor mutational burden (TMB) of all analyses.** (A) Scatter plot displaying TMB of each primary (red) and recurrent (blue) tumor in the NOT cohort. The red and blue lines indicate the median TMB value of the primary and recurrent tumors, respectively. (B) Boxplot displaying the distribution of TMB value per molecular subset. (C) Scatter plot displaying TMB values of all the TCGA cohorts and the NOT cohort. Red horizontal lines indicate median TMB. Cohort sizes are indicated on top. The NOT diffuse glioma cohort and the TCGA glioma cohorts are highlighted.

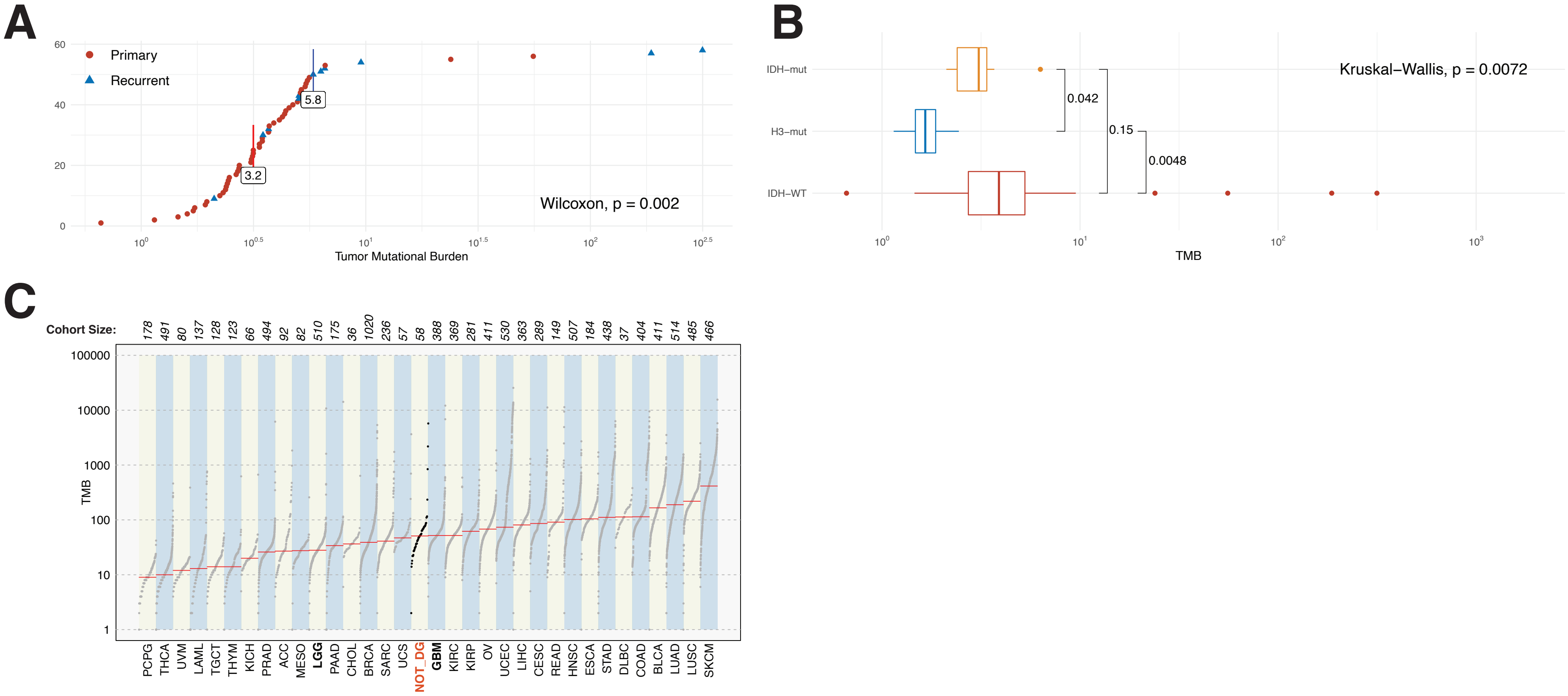

**Figure S4: The overall pattern of SCNAs by cytoband.** The vertical axis represents the genome. The horizontal axis indicates the frequency of deletions (blue) and amplifications (red). Selected consensus tumor suppressor genes/oncogenes located within corresponding chromosomes are indicated.

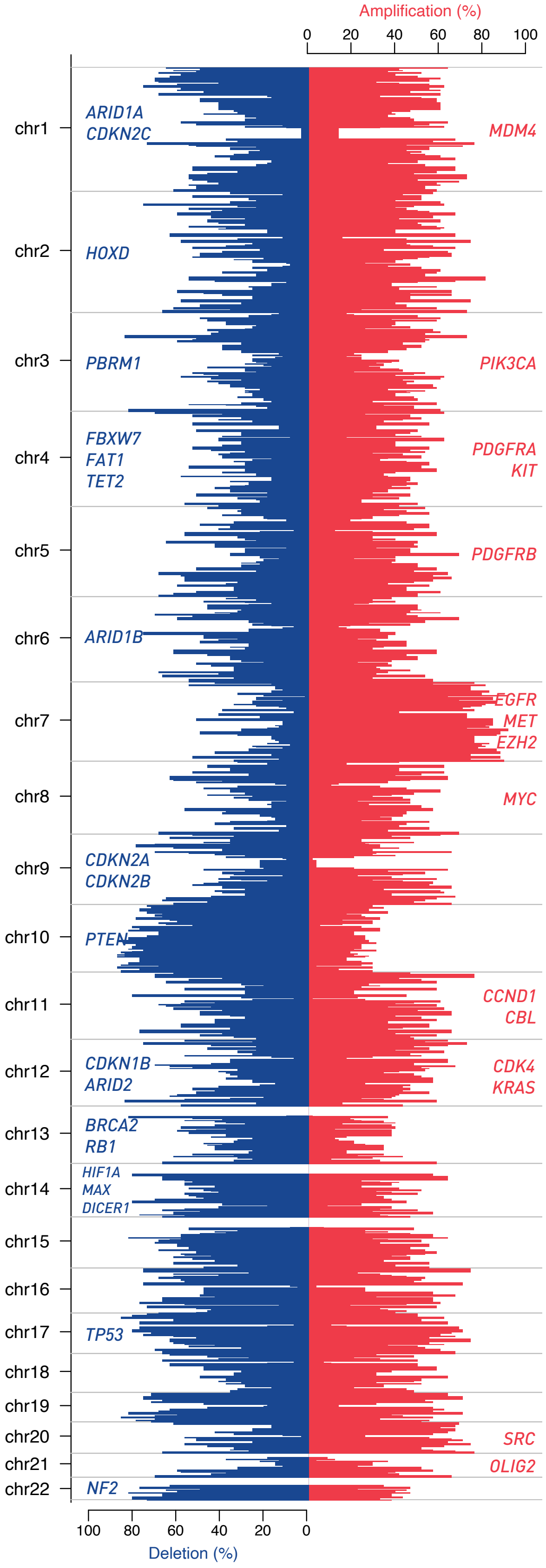

Supplement: Supplementary file 2 — Additional file 2. Supplementary figures and tables. [file 12920_2021_904_MOESM2_ESM.pdf]
